# NEXMIF overexpression is associated with autism-like behaviors and alterations in dendritic arborization and spine formation in mice

**DOI:** 10.3389/fnins.2025.1556570

**Published:** 2025-06-18

**Authors:** KathrynAnn Odamah, Mauricio Toyoki Nishizawa Criales, Heng-Ye Man

**Affiliations:** ^1^Department of Biology, Boston University, Boston, MA, United States; ^2^Department of Pharmacology, Physiology & Biophysics, Boston University School of Medicine, Boston, MA, United States; ^3^Center for Systems Neuroscience, Boston University, Boston, MA, United States

**Keywords:** Autism Spectrum disorder, X-linked intellectual disability (XLID), overexpression, mouse behavior, NEXMIF gene, NEXMIF duplication, neuronal deficits

## Abstract

We previously found that loss of the X-linked gene NEXMIF results in ASD and intellectual disability (ID). Duplication of chromosomal segments containing NEXMIF has been associated with ASD/ID in humans, but the direct link to the NEXMIF gene, as well as the behavioral and cellular consequences of NEXMIF overexpression, have not yet been explored. Here, we developed a lentivirus containing the human NEXMIF gene which was bilaterally injected into the ventricles of newborn mice. At adolescent ages, the mice were subjected to various behavioral assays to assess the presence of ASD-like behaviors and comorbidities, followed by the collection of brain tissue to examine changes in neuron morphology, protein expression, and the transcriptome. We report that NEXMIF overexpression in mice led to impaired communication, short-term memory deficits, reduced social behavior, hyperactivity, repetitive/restrictive behaviors, anxiety-like behavior, and altered nociception at adolescent ages, accompanied by attenuated dendritic spine density. RNA sequencing revealed that elevated NEXMIF dosage leads to strong dysregulation in the expression of genes involved in synaptic transmission, neuron differentiation, and post-synaptic membrane potential. Moreover, cultured neurons overexpressing NEXMIF displayed increased dendritic arborization. These findings indicate that NEXMIF overexpression results in transcriptional and cellular deficits that contribute to the development of ASD-like behaviors.

## Introduction

Autism spectrum disorders (ASDs) are characterized persistent deficits in social communication and social interaction and the tendency for repetitive/restricted behaviors and (CDC, 2024). ASD is becoming increasingly devastating due to its high prevalence, affecting 1 in 36 children (8-year-olds) in the United States (CDC, 2024). ASD is often comorbid with several medical and psychiatric conditions, such as epilepsy, gross and fine motor deficits, attention-deficit/hyperactivity disorder (ADHD), and intellectual disability (ID; Bougeard et al., 2021; Khachadourian et al., 2023). A number of ASD-associated genes are located on the X chromosome, and several of these genes have been linked to the development of X-linked intellectual disability (XLID), particularly in males (Nguyen et al., 2020; Piton et al., 2013; Wang et al., 2023). In our previous work, we found that patients with a loss of expression of the X-linked gene *NEXMIF* (also known as *Xpn*, *KIAA2022*, or *KIDLIA*) display ID and ASD characteristics, including repetitive behaviors, social deficits, and a limited or lack of language capability accompanied by seizures and microcephaly (Van Maldergem et al., 2013). *NEXMIF* has since been listed as a Score 1/Category 1 ASD gene in the SFARI database and is now commonly tested in clinics for genetic diagnosis of ASD and XLID (Van Maldergem et al., 2013; Cantagrel, 2004; Cantagrel et al., 2009; de Lange et al., 2016; Stamberger et al., 2021).

*NEXMIF* is located at Xq13.3 and encodes a large ~170 kilodalton protein, which is strongly localized to the nucleus of neurons and highly expressed in the developing fetal brain, adult cerebral cortex, hippocampus, cerebellum, and olfactory bulb (Cantagrel, 2004; Cantagrel et al., 2009). At the structural level, the NEXMIF protein contains a domain of unknown function (DUF4683) which has sequence similarity to REV3L, the catalytic subunit of DNA polymerase zeta (Wittschieben et al., 2000), and to the transcription factor Gibbin, which regulates the expression of mesoderm genes during early development (Collier et al., 2022). Currently, the cellular function of *NEXMIF* is not well understood; however, early studies have implicated NEXMIF in the regulation of neural circuit formation during early development, as well as the regulation of cytoskeletal dynamics involved in neurite outgrowth (Ishikawa et al., 2012; Magome et al., 2013). Furthermore, NEXMIF was recently found to regulate the expression of genes responsible for *β*-cell proliferation in the pancreas, as well as the suppression of retrotransposon LINE1 activity in the brain, pancreas, and testes in CRISPR/Cas9-generated *Nexmif* knockout (KO) mice (Stekelenburg et al., 2022). Consistent with these findings, we have previously shown that loss of *Nexmif* leads to defects in neuron migration, dendrite growth, and synapse formation and function (Van Maldergem et al., 2013; Gilbert et al., 2020; Gilbert and Man, 2016; O’Connor et al., 2024). At the behavioral level, our in-house generated *Nexmif* KO male mice show hyperactivity, deficits in learning/memory function, and typical ASD-like features including repetitive overgrooming, restrictive marble burying, impairments in social behavior, and altered communication, accompanied by frequent seizures at mature ages (Gilbert et al., 2020). In addition, consistent with clinical reports in females (de Lange et al., 2016; Stamberger et al., 2021; Athanasakis et al., 2014; Badura-Stronka et al., 2023; Chen et al., 2022; Cioclu et al., 2021; Farach and Northrup, 2016; Langley et al., 2022; Moysés-Oliveira et al., 2015; Palmer et al., 2020; Wang et al., 2023; Webster et al., 2017), we find that our *Nexmif* heterozygous (HET) female mice, with an approximately 50% reduction in NEXMIF expression, also demonstrate ASD-like behaviors, hyperactivity, memory impairments, and seizures (O’Connor et al., 2024), indicating high sensitivity to NEXMIF dosage during brain development. Taken together, these cellular and behavioral findings suggest that NEXMIF plays a crucial role in transcriptional regulation and early brain development, and its loss or change in abundance is associated with neuronal impairments, intellectual deficits, and ASD-like behaviors in mice.

Recent clinical reports have demonstrated duplication of the *NEXMIF* gene in humans (Palmer et al., 2020; Charzewska et al., 2015; Mehvari et al., 2020). Using whole genome sequencing, one study identified 14 males from 9 families with duplications of the Xq13.2-q13.3 region, in which the *NEXMIF* gene resides. These duplications were associated with ID, autistic features, hyperactivity, microcephaly, and common abnormal facial features in all males (Palmer et al., 2020). Duplication of Xq13.2-q13.3 was also reported in a multi-generational Iranian family consisting of seven affected males presenting with ID, physical abnormalities, anxiety, impaired social interactions, and speech deficits (Mehvari et al., 2020). These findings suggest an implication of *NEXMIF* in the observed behavioral phenotypes. More direct evidence comes from a study reporting that duplication of the entire *NEXMIF* locus (dupXq13.3) was associated with varying degrees of ASD/ID severity in five affected males of a multi-generational Polish family (Charzewska et al., 2015). However, because the Xq13.2-q13.3 duplication affects multiple genes, such as *RLIM*, *FTX* and *SLC16A2*, and NEXMIF protein expression in the brain has not been studied in human patients, it is unclear as to whether *NEXMIF* gene duplication and protein hyperexpression indeed lead to ASD/ID symptoms. To address this, we overexpressed the human *NEXMIF* gene in the mouse brain via intracerebroventricular (ICV) brain injections at postnatal day 1 (P1) and conducted behavioral tests for ASD-like phenotypes and biochemical and morphological analyses of neurons. We find that mouse brains of NEXMIF viral injection (NEX^OX^ mice) show a significant increase in NEXMIF mRNA and protein levels. Importantly, NEX^OX^ mice demonstrate altered communication at neonatal ages, and display impaired object recognition memory, lack of social novelty preference, hyperactivity, anxiety, altered thermal nociception, and repetitive/restrictive behaviors at adolescent (P30-P70) ages. Additionally, NEXMIF overexpression leads to aberrance in dendritic growth and spine formation, as well as dysregulation of genes involved in synaptic function, neuron differentiation, memory, receptor signaling, and behavior. These findings provide direct evidence supporting that NEXMIF hyper-expression leads to impairments in brain development, as well as cognitive and social behaviors.

## Materials and methods

### Animal care and use

All the procedures involving animal use followed the policies of the Institutional Animal Care and Use Committee (IACUC, PROTO201800574) at Boston University (BU). Mouse colonies were maintained on a C57BL/6 J genetic background in the Animal Science Center (ASC) at the BU Charles River Campus. Littermate male and female mice aged postnatal (P) day 5–9 or 30–70 were used for all behavioral experiments. A subset of the animals behaviorally tested at P30 was tested again at P60. Due to there being no effect of sex on our findings, male and female data were combined at each timepoint. All experiments were conducted during the light phase. Following all *in vivo* injection experiments and behavioral tests, IACUC-approved euthanasia methods were used to sacrifice mice prior to tissue collection, specifically via CO_2_ inhalation at a flow rate of 4 liters/min in the home-cage for 3 min. Cervical dislocation was performed 2 mins after cessation of breathing.

### Primary neuron culture

Cortical brain tissue was dissected from E18 rat fetus brains of either sex and prepared for primary culture. Brains were digested in a digestion buffer [papain (15 mg/mL in Hanks balanced salt solution, Sigma-Aldrich #4762), L-Cysteine (4 mg/mL in Hanks balanced salt solution, Sigma-Aldrich #C7352), and 0.5 M EDTA pH 7.0] for 20 min at 37°C, then triturated in a trituration buffer [0.1% DNase (Thermo Fisher #PA5-22017), 1% ovomucoid (Sigma-Aldrich #T2011)/1% bovine serum albumin (Sigma-Aldrich #05470) in Dulbecco’s modified Eagle’s medium (DMEM)] to fully dissociate neurons. Dissociated neurons were counted and plated on 18-mm circular coverslips (Carolina #633013) in 60-mm Petri dishes (five coverslips/dish) and 6-well culture plates that had been coated in poly-l-lysine (Sigma-Aldrich #P2636; 100 μg/mL in borate buffer) overnight at 37°C then washed three times with sterile deionized water and left in plating medium [minimal essential medium (500 mL) containing 10% fetal bovine serum (Atlanta Biologicals #S11550), 5% horse serum (Atlanta Biologicals #S12150), 31 mg L-cysteine, 1% penicillin/streptomycin (Corning #30-002-Cl), and 1% L-glutamine (Corning #25-005-Cl) before cell plating]. The day after plating, plating medium was replaced by feeding medium (Neurobasal medium supplemented with 1% horse serum, 2% SM1, and 1% penicillin/streptomycin and 1% L-glutamine) which was supplemented with 5′-fluoro-2′-deoxyuridine (10 μm; Sigma-Aldrich #F0503) after 7 d *in vitro* to suppress glial growth.

### Plasmids

A transgene for human *NEXMIF* was cloned into the FUW lentiviral expression vector, and the resulting FUW-*NEXMIF* plasmid was packaged into a lentivirus. As a control, the empty FUW vector was packaged into a separate lentivirus. FUW was a gift from David Baltimore (Addgene plasmid #14882). All unique/stable reagents generated in this study are available from the lead contact without restriction.

### Virus preparation

Lentiviruses (LV) were produced by co-transfecting HEK293T cells with the DNA constructs and viral packaging and envelope proteins (pRSV/REV, pMDLg/pRRE, and pCMV-VSV-G) using polyethylenimine reagent (Polysciences #23966). Sodium pyruvate (Thermo Fisher #11360070) was added to the medium 24 h later to supplement the cells. Conditioned medium containing the viral particles was harvested 48 h later and filtered through a 0.45-μm filter. PEG-it Virus Precipitation Solution (System Biosciences # LV810A) was added to the medium, and the mixture was left to incubate at 4°C for 72 h. The mixture was centrifuged at 1500 g for 30 min at 4°C and the viral pellet was resuspended in sterile 1X phosphate buffered saline (PBS). The virus was divided into 10 uL aliquots and stored at −80°C. The LV packaging constructs were gifts from Didier Trono (Addgene plasmids #12251 and #12253) and Bob Weinberg (Addgene plasmid #8454).

### Neuron transduction

For viral transductions, rat cortical neurons plated on coverslips or 6-well plates were infected with LV either at 3 days *in vitro* (DIV 3) or at DIV 10. 24 h later, the medium was replaced with fresh medium, and the cells were incubated at 37°C in a 5% CO_2_ incubator for the desired length of time.

### Mouse brain injections

For intracerebroventricular (ICV) injections of LVs, postnatal day 1 (P1) C57/BL6 male and female mouse pups were cryo-anesthetized on wet ice for 3 min prior to bilateral ventricular injection (1.13×10^6^ viral particles per ventricle) on a chilled stage using a 10 μL syringe with a sterile 32-gauge needle (Hamilton #7653–01). Fast Green dye (1 μL) was added to the 10 μL virus aliquots to visualize and confirm successful injection. Following injection, pups were warmed on an isothermal heating pad with home-cage bedding before being returned to the dam.

### Immunocytochemistry of cultured neurons

Cortical neurons were fixed for 8 min in a 4% paraformaldehyde (PFA) / 1X PBS solution at room temperature (RT). Cells were rinsed two times in 1X PBS followed by membrane permeabilization for 10 min in 0.3% Triton-X-100 (Sigma Aldrich #T8787) in 1X PBS. Cells were then rinsed two times in 1X PBS followed by incubation with primary antibodies overnight at 4°C, washed three times with cold 1X PBS, and incubated with Alexa Fluor-conjugated fluorescent secondary antibodies (1:500, Thermo Fisher) for 1 h at RT. Cells were then washed three times with cold 1X PBS, with the first wash containing Hoechst (1:10,000, Thermo Fisher #62249) and mounted to microscopy glass slides with Prolong Gold antifade mounting reagent (Thermo Fisher #P36930) for subsequent visualization. Mounted coverslips were kept overnight in the dark at RT before imaging.

### Golgi staining

Whole brains from P40 mice were subjected to Golgi neuron staining using the FD Rapid GolgiStain Kit (FD Neurotechnologies #PK401) according to the manufacturer’s instructions. Mice were sacrificed in a 4% CO_2_ chamber and brains were collected and rinsed in ice-cold PBS. Brains were immersed in a Golgi-Cox solution containing potassium dichromate, mercuric chloride, and potassium chromate. The solution was replaced after 24 h of immersion with fresh solution and stored at RT in the dark for 2 weeks. After immersion, the brains were placed in Tissue Freezing Medium (Electron Microscopy Sciences #72592), rapidly frozen in a dry ice/methanol bath, and stored at −20°C prior to slicing. Brain slices were sectioned coronally at 100 μm thickness on a cryostat and were mounted on gelatin-coated slices (FD Neurotechnologies #PO101) and air dried at RT before further processing. Sections were then rinsed in distilled water, incubated in staining solution for 10 min, and dehydrated with 50, 75, 95%, and finally 100% ethanol. Sections were defatted in xylene and mounted onto coverslips with Permount mounting medium (Fisher Scientific # SP15-100). Sections were stored at RT in the dark for 3 weeks prior to visualization.

### Western blot

Brains were dissected on ice immediately after sacrificing animals at the appropriate time points. For a ∼ 30 mg piece of cortical tissue, or 1×10^6^ primary cortical neurons, ∼750 μL of ice-cold lysis buffer [50 mM Tris–HCl pH 8, 150 mM NaCl, 1% Triton X-100, 0.5% sodium deoxycholate (SDOC), 0.1% sodium dodecyl sulfate (SDS), supplemented with 100X protease inhibitor cocktail (Apex Bio #K1011)] was added and samples were homogenized mechanically with a pestle, followed by sonication (for 10 s). Samples were then centrifuged for 15 min at 13,000 rpm at 4°C in a microcentrifuge. The tubes were placed on ice and the supernatant was carefully aspirated and placed into a fresh tube kept on ice. Samples were subjected to a BCA assay according to the manufacturer’s protocol (Thermo Fisher #23225) to determine protein concentrations. Protein levels were normalized with the lysis buffer, and an equal volume of 2x sample reducing buffer [5% SDS, 150 mM Tris–HCl pH 6.8, 0.05% Bromophenol Blue (Sigma-Aldrich #B0126), 5% fresh 2-mercaptoethanol (Sigma-Aldrich #M3148)] was added to the samples. The lysates were then boiled for 10 min at 95°C to prepare for SDS-PAGE.

SDS-PAGE was performed to separate proteins of interest using standard procedures. Samples were run on 6–12% gels at 110 V for 1 h. Proteins were transferred at 150 mA overnight to PVDF membranes and blocked for 1 h in 5% bovine serum albumin (BSA, Sigma Aldrich #A2153) prepared in 1X tris-buffered saline supplemented with 0.1% Tween (TBST). Following block, membranes were probed with the appropriate primary antibody diluted in Signal Enhancer Hikari Buffer (Nacalai United States #NU00101) overnight at 4°C. Membranes were washed 3× 5 min each in 1X TBST and then incubated with the appropriate secondary antibody for 1 h. After secondary incubation, membranes were washed 3X with 1X TBST. Blots were visualized using the Azure Radiance Plus chemiluminescence detection system (Azure Biosystems #AC2103) on the Sapphire Biomolecular Imager (Azure Biosystems) and analyzed using NIH Fiji (RRID: SCR_002285).

### Antibodies

Primary antibodies to the following proteins were used: rabbit anti-KIAA2022 [1:300 (cultured neurons for ICC), Sigma #HPA000407], rabbit anti-KIAA2022 (1:750 for WB; Biorbyt Orb312213), rabbit-anti BAIAP3 (1:1000 for WB, Synaptic Systems 256,003), rabbit-anti SGK1 (1:1000 for WB; Cell Signaling Technology #12103), rabbit-anti Cerebellin-1 (1:1000 for WB; Boster Bio A09176-1), rabbit anti-*β*-tubulin III (1:1000 for WB, Sigma-Aldrich T2200), and rabbit anti-*α*-tubulin (1:1000 for WB, Cell Signaling Technology #2144).

The following secondary antibodies were used: IgG-HRP for WB [1:10000; Thermo Fisher, mouse (#62–6,520) and rabbit (#31460)], and Alexa Fluor 488 (1:500, Thermo Fisher, mouse: #A32723; rabbit: #A32731) and Alexa Fluor 555 (1:500, Thermo Fisher, mouse: #A32727; rabbit: #A32732) for ICC. For ICC, all primary and secondary antibodies were diluted at the above concentrations in IHC-Tek™ Antibody Diluent pH 7.4 (IHCWorld, #IW-1000).

### Reverse transcription and quantitative polymerase chain reaction

Total RNA was extracted from either 1×10^6^ primary cortical neurons or ~30 mg of mouse cortical tissue using the RNeasy Mini kit (Qiagen #74106). RNA concentrations were diluted with nuclease-free water (NEB #B1500S) to 500 ng (primary neurons) or 1 ug (brain tissue), followed by reverse transcription to cDNA using the EasyQuick RT MasterMix (CoWin Biosciences #CW2019). 1 uL of the cDNA was added to the HotStart™ Universal 2X SYBR Green qPCR Master Mix (ApexBio Technology #K1170) in a 20 μL volume and real-time fluorescence quantitative PCR was performed using the 7900HT Fast Real-Time PCR System (Applied Biosystems) to detect mRNA levels of human *NEXMIF* and *β-actin* with the appropriate primers. The primers used: *NEXMIF* (F: tgatcctggtcgtgcaaaca R: ttgtggacctgttctcgctc); *β-actin* (F: cattgctgacaggatgcagaagg R: tgctggaaggtggacagtgagg). Target gene expression was normalized to *β-actin* prior to using the delta delta Ct method to determine the fold change in gene expression in response to lentiviral expression of NEXMIF.

### RNA sequencing

Hippocampal brain tissue collected from a total of 4 mice (2 CTRL vs. 2 NEX^OX^) were preserved in RNA*later*™ Stabilization Solution (Invitrogen #AM7020) prior to being sent out for commercial RNA-sequencing services provided by GENEWIZ from Azenta Life Sciences. RNA-seq datasets were generated and analyzed by GENEWIZ to identify differentially expressed genes and Gene Ontology (GO) Enrichment Analysis was conducted using the PANTHER 19.0 classification system. All DEGs had an adjusted *p*-value < 0.05 and at least a 1.3-fold change in expression.

### Ultrasonic vocalization recording

Ultrasonic vocalizations (USVs) were recorded from mouse pups at postnatal (P) days 5, 7, and 9 using a CM16/CMPA microphone (Avisoft Bioacoustics, Berlin, Germany). Briefly, a pup was separated from their dam and littermates to illicit calling and then isolated in a recording chamber with the microphone 15 cm above the chamber. Between each pup, the recording chamber was cleaned with 70% ethanol and dried. The pups were recorded for 5 min in a 16-bit format at a sampling rate of 300 kHz. The microphone was connected to a pre-amplifier UltraSoundGate 116Hb (Avisoft Bioacoustics, Berlin, Germany) and the digitized sonograms were stored in a computer. The recordings were analyzed using DeepSqueak (Coffey et al., 2019). The number of calls, the total time spent calling, the mean call duration, and the peak frequency were measured.

### Three-chamber social test

A three-chambered box measuring 65 × 28 × 28 cm was constructed from 0.75 in thick white plastic board with 4 × 4 in cut-out doors in the walls to the center chamber allowing movement between chambers. A small wire cage was placed in each of the side chambers to later house stranger mice. Test mice were habituated to the apparatus with empty cages in both side chambers and allowed to move freely between all three chambers for 5 min on days 1 and 2. On the testing day (day 3), the side doors were blocked with white plastic boards and test mice were singly placed into the center chamber with an age- and sex-matched stranger mouse (Mouse 1) placed under the wire cage in either of the side chambers. Once the doors were unblocked, the test mouse was allowed to move freely within the apparatus for 5 min. The test mouse was then returned to the center chamber, the doors were blocked again, and a second age- and sex-matched mouse (Novel Mouse) was placed in the wire cage on the other side chamber. The center doors were un-blocked again, and the test mouse was allowed to move freely within the apparatus for 5 min. Animals’ movement was analyzed for the time spent interacting with each mouse or empty cage (nose ≤ 2 cm). Between test mice, the entire apparatus was wiped with 70% ethanol to eliminate odor cues.

### Novel object recognition test

The novel object recognition (NOR) test was conducted in an arena constructed from a 0.75 in-thick white plastic box measuring 28 × 28 × 28 cm. Mice were habituated to the environment for 5 min on days 1 and 2. On the test day (day 3), two identical objects (25 cm2 cell culture bottles filled with fresh bedding) were placed diagonally from each other on the floor of the box environment. Each mouse was singly placed into the center of the box and allowed to freely explore the two objects for 10 min. Following the exploration, mice were placed back into their home cage. Four hours later, one of the identical objects was replaced with a salient, “novel” object (10 × 5 × 5 cm LEGO® block) and placed diagonally from the “familiar” object. Each mouse was again singly placed into the center of the box and allowed to freely explore the two objects for 5 min. Movement was analyzed for the time spent interacting with each object during each session (nose ≤ 2 cm). Between test mice, the entire apparatus was wiped with 70% ethanol to eliminate odor cues.

### Open field test

The Open Field test was conducted in the same arena as the NOR test. Lights in the testing room were dimmed for comfort of the animal with only a small desk lamp in the corner for the experimenter. Each mouse was singly placed into the center of the arena and allowed to freely explore for 5 min. Animals’ movement was analyzed for the movement speed, total distance traveled, and time spent in the center of the arena.

### Grooming test

Grooming was assessed in the animal’s home cage with a 3 in thick layer of fresh pine chip bedding. Mice were briefly moved into a holding cage prior to being singly placed into their home cage and allowed to move freely for 20 min. Video recordings were captured from the side of the cage. The amount of time spent grooming and the number of grooming events during the last 10 min of the session were manually quantified. Grooming was considered as licking of the paws followed by cleaning of the snout, eyes, whiskers, body, and tail with backward and upward sweeps.

### Elevated zero maze

Mice were singly placed on a circular track that was elevated 2 ft. above the ground. Two sides of the track had closed arms, while the rest of the track was open. A camera was placed ~5 feet above the track to record animals’ movements. The test mouse was placed at an open/closed arm boundary facing toward the closed arm. The mouse was allowed to freely explore the open and closed arms of the track for 10 min. The amount of time spent in the open arms relative to the total distance traveled was recorded as a measure of anxiety.

### Light dark box test

The Light Dark Box test was conducted in a 0.75 in-thick black plastic box measuring 28 × 28 × 28 cm. The box was divided by a thin black cardboard into 2 sections: a “light” compartment (2/3^rd^ of the box) exposed to an overhead bright light, and a “dark” compartment (1/3^rd^ of the box) shielded from light by a thick black piece of cardboard. A camera was placed ~3 feet above the box to track animals’ movements. The test mouse was singly placed into a corner of the light compartment, and a 3 × 3 in. opening in the center of the cardboard divider allowed the mouse to move freely between the two compartments. The amount of time spent in the “light” compartment relative to the dark compartment was recorded over a 10-min session as a measure of anxiety.

### Marble burying test

The marble burying test was conducted on P60 mice only in a square plastic bin with a 3 in thick layer of fresh pine chip bedding. Sixteen shiny glass marbles (0.25 in diameter) were arranged in a 4 × 4 grid on top of the bedding. Mice were singly placed into the bin and allowed to move freely and bury marbles for 25 min. The number of marbles buried after 5, 10, 15, 20 and 25 min were manually counted.

### Hot plate assay

Mice were habituated to a room temperature hot plate under a cylindrical glass container 5 min / day for 2 days. On the third day, the hot plate was set to 55°C. The test mouse was placed on the hot plate and covered with the glass container, and the amount of time that passed until the mouse licked its hind paw or front paws (latency to withdraw) was recorded.

### Microscopy

Exposure time for the fluorescence signals was adjusted manually so the signals were within a full dynamic range. Once the parameters were set, they were fixed and used throughout image acquisition for each experiment.

For ICC: Fluorescent images were collected with a 63x oil-objective on a ZEISS Axio Imager Z2 Upright Microscope using the ZEISS ZEN software. Neuron images were quantified using NIH ImageJ software.

For Golgi staining: images were acquired using brightfield transmitted light with a 63x oil-objective on a ZEISS Axio Imager Z2 Upright Microscope using the ZEISS ZEN software. The number and density of apical and basal spines on neurons were measured with Fiji (ImageJ; Schindelin et al., 2012) software using the Dendritic Spine Counter plugin (Image J wiki, 2021).

### Sholl analysis

Complexity of dendritic arborization was quantified using Fiji software. The dendrites of each neuron were manually traced, and the tracings were used to obtain measurements of the number of dendrites, total dendritic length, mean dendritic length, and dendritic complexity evaluated by Sholl analysis. The Sholl analysis plugin within Fiji measures dendritic branching complexity by counting the number of intersections between the dendritic filaments and concentric circles set at 40 μm intervals from the soma. The Sholl value at each radius was then plotted versus the radius of its intersecting circle. Neurons were imaged and randomly selected for analysis regardless of cell-type (i.e., excitatory vs. inhibitory).

### Statistical analysis

For ICC and Sholl Analysis: A two-tailed student’s t test was used to determine significant differences in nuclear intensity, average dendritic length, total dendritic length, and the average number of dendrites between control virus- and NEXMIF virus-treated neurons. An unpaired Multiple t test was used to determine significant differences in the average number of crossings (Sholl) at each stepwise distance from the soma between control virus- and NEXMIF virus-treated neurons. An area under the curve (AUC) analysis was performed for the Sholl curves of control virus- and NEXMIF virus-treated neurons and a two-tailed student’s t test was used to determine significant differences between the AUC of the Sholl curves.

For Golgi: A two-tailed student’s t test was used to determine significant differences in spine density between P40 CTRL and NEX^OX^ mice.

For Ultrasonic Vocalization (USV): For analysis of USV data, a Two-Way ANOVA with Tukey’s *Post Hoc* test was used to determine significant differences in the average number of calls, the total number of calls, and the average call duration between CTRL and NEX^OX^ pups at P5, P7, and P9. For the analysis of the average number of calls per minute at each age timepoint, a Simple Linear Regression with Equal Slopes test was used to determine significant differences between slopes of the CTRL vs. NEX^OX^ graphs.

For Behavioral Tests: Video recordings were captured with a Logitech c920 webcam during each test. Locomotion tracks were generated using the Mouse Activity Analyzer in MATLAB software (Zhang et al., 2020). These tracks were to quantify the desired aspects of the animals’ movement. For the Elevated Zero Maze and Light Dark Box tests, DeepLabCut (Mathis et al., 2018) was used to extract precise coordinates of animal movement, which were subsequently analyzed using custom MATLAB scripts (Fournier et al., 2024). Either a two-tailed student’s t test or a Two-Way ANOVA with Tukey’s or Bonferroni’s *Post Hoc* Test was used to determine significant differences in behavior between the two test groups: CTRL and NEX^OX^.

Experimenters were not blinded to the experimental conditions; however, standardized protocols were used for mouse handling, behavioral testing, and scoring. Testing conditions, such as the time of day, location, noise and lighting, were kept consistent, and the same experimenter was used for all tests to reduce bias. The Novel Object Recognition test, Three-Chamber Social test, Marble Burying test, Grooming, and Hot Plate assay were manually scored. Ultrasonic Vocalization, Open Field, Elevated Zero Maze, and Light Dark Box tests were scored unbiasedly in MATLAB. All data are expressed as mean ± SEM and were analyzed using GraphPad Prism 10 statistical software (GraphPad Software, Boston, MA). *p* < 0.05 is considered statistically significant. *p* values are presented as *p* > 0.05 (ns, not significant), *p < 0.05, **p < 0.01, ***p < 0.001, and ****p < 0.0001.

## Results

### Postnatal intraventricular brain injection of LV-NEXMIF increases NEXMIF expression in mice

**Figure 1:**
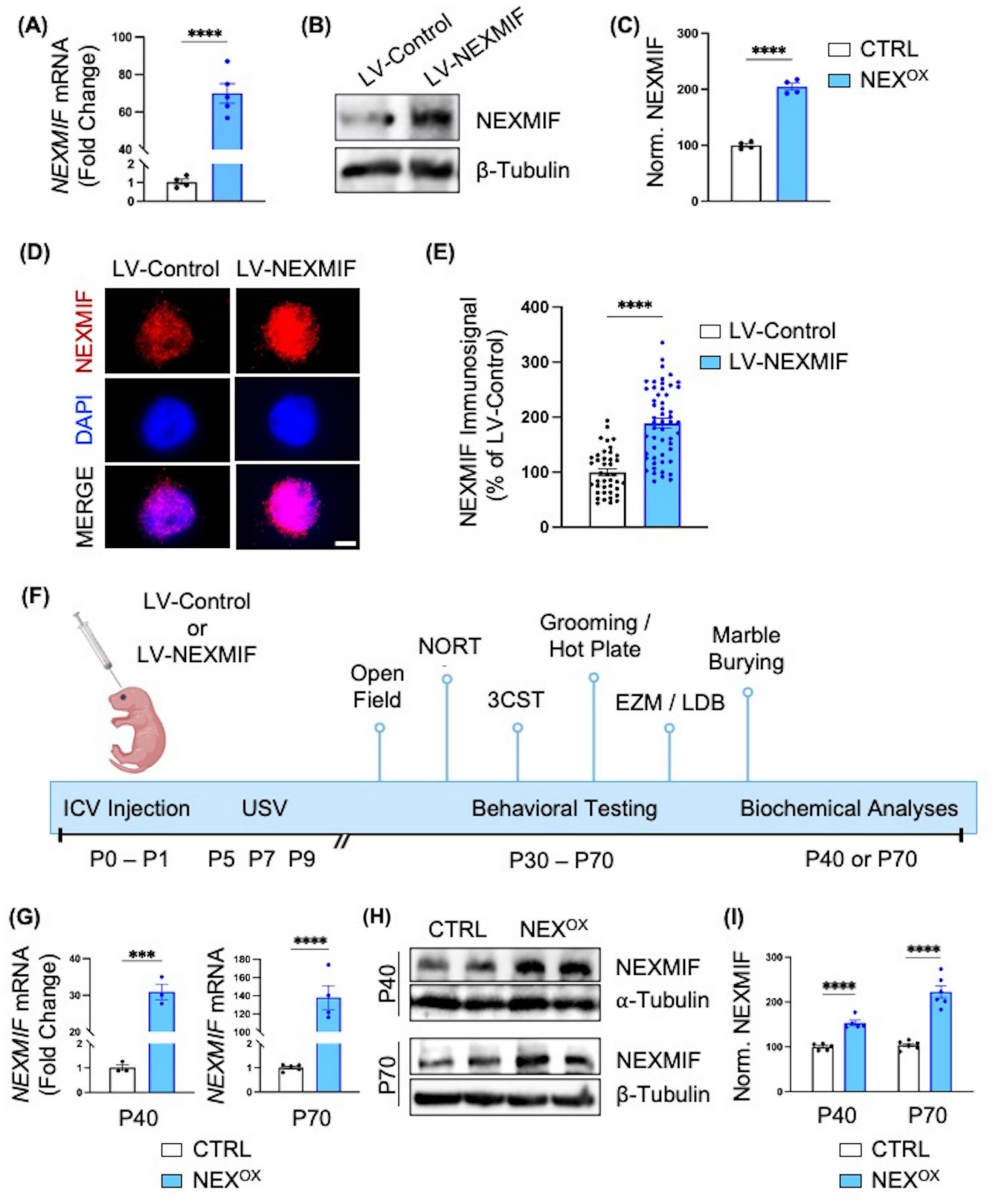
Intraventricular brain injection of LV-NEXMIF at P1 increases NEXMIF expression in adolescent mice. **(A)** Cultured rat cortical neurons were infected with LV-NEXMIF at 10 days *in vitro* (DIV) and cells were lysed for RNA extraction and reverse transcription into cDNA at DIV 17. Viral NEXMIF mRNA was significantly increased over ~70-fold in LV-NEXMIF-treated neurons, relative to neurons infected with FUW control lentivirus (LV-Control). **(B,C)** Western blot from DIV 17 neuronal lysates **(B)** and quantification **(C)** showing that NEXMIF protein was also significantly increased in LV-NEXMIF-treated neurons, relative to control. **(D)** Representative images showing NEXMIF immunostaining (top row) merged with DAPI nuclear counterstain (bottom row) from LV-Control-treated (left panel) and LV-NEXMIF-treated (right panel) neurons at DIV 17; Scale bar = 5 μm. **(E)** NEXMIF nuclear intensity was significantly increased in LV-NEXMIF-treated neurons, relative to control. **(F)** Schematic illustration of the experimental timeline. LV-Control or LV-NEXMIF virus was bilaterally injected into the ventricles of male and female C57BL/6 mouse brains at postnatal (P) days 0–1. The two injected groups were: LV-Control (CTRL) and LV-NEXMIF (NEX^OX^) mice. Beginning at P30 or P60, injected mice were subjected to a series of behavioral tests to examine the effect of NEXMIF overexpression on behavior using test that detect the presence of autistic-like phenotypes [Communication (USV), Grooming/Marble Burying, Sociability and Social Novelty Preference], hyperactivity and anxiety [Open Field Assay, Elevated Zero Maze (EZM), and Light/Dark Box (LDB)], sensory deficits (Hot Plate Assay), and short-term memory impairment [Novel Object Recognition test (NORT)]. Following behavioral testing, mice were sacrificed and perfused for brain slice immunostaining, or brain tissue was collected for mRNA/protein quantification, Golgi staining for spine morphology, or RNA sequencing. **(G)** Real-time (RT)-qPCR analysis confirmed significantly increased expression of human NEXMIF mRNA in cortical brain tissue from P40 and P70 NEX^OX^ mice, relative to CTRL mice. **(H)** Western blot of NEXMIF and β-tubulin protein expression in the medial prefrontal cortex (mPFC) of P40 and P70 NEX^OX^ mice. **(I)** Quantification of the western blots in **(H)** normalized to β-tubulin loading control revealed significantly increased expression of NEXMIF protein in the mPFC of NEX^OX^ mice, relative to CTRL mice. Data are represented as average ± SEM. Two-tailed student’s *t* test **(A,C,E,G,I)**. ***p < 0.001; ****p < 0.0001. ns, Not significant. Figure 1F created with Biorender.com.

We previously generated *Nexmif* knockout (KO) male and heterozygous (HET) female mouse models which demonstrate ASD-like phenotypes (repetitive behaviors, impaired communication, and altered social preference), accompanied by the presence of anxiety, restrictive behaviors, and deficits in learning and memory at adult ages (Gilbert et al., 2020; O’Connor et al., 2024). Conversely, several studies have also modeled human ASDs due to gene overexpression in mice, such as *Mecp2, Fmr1*, and *Ube3a* duplication, which have been associated with similar neuronal and autistic-like behavioral deficits (Ash et al., 2021; Collins et al., 2004; Collins and Neul, 2022; Krishnan et al., 2017; Mononen et al., 2007; Peier et al., 2000; Rio et al., 2010; Roy et al., 2023; Smith et al., 2011; Tian et al., 2024; Vengoechea et al., 2012). Given the recent reports of *NEXMIF* duplication in ASD patients (Palmer et al., 2020; Charzewska et al., 2015; Mehvari et al., 2020), we wanted to determine whether NEXMIF overexpression could indeed induce ASD-like phenotypes in mice, as well as investigate the underlying mechanisms. To this end, we generated a lentivirus containing a human *NEXMIF* transgene construct driven under the human ubiquitin C (hUbC) promoter. To assess the efficacy of the NEXMIF lentivirus (LV-NEXMIF), cultured rat cortical neurons were infected with either FUW control lentivirus (LV-Control) or LV-NEXMIF at 10 days *in vitro* (DIV) and the cells were either immunostained or lysed for mRNA and protein quantification at DIV 17. RT-qPCR analysis revealed that viral *NEXMIF* mRNA was significantly increased over ~70-fold in LV-NEXMIF-treated neurons, relative to LV-Control neurons (Figure 1A). Consistently, western blot quantification and immunostaining showed that NEXMIF immunosignal was also significantly increased in LV-NEXMIF-treated neurons (Figures 1B–E; Supplementary Figure 1A).

To confirm the expression of LV-NEXMIF in mice, we performed bilateral intracerebroventricular (ICV) injections of LV-Control (CTRL) or LV-NEXMIF (NEX^OX^) in postnatal (P) day 1 C57/BL6 mice (Figure 1F). Prior to behavioral testing, we recorded the bodyweights of CTRL and NEX^OX^ mice, as well as assessed their motor control using the rotarod test. We observed no differences in bodyweight between CTRL and NEX^OX^ mice (Supplementary Figure 2A), and found that NEX^OX^ mice show no impairments in motor coordination (Supplementary Figures 2B,C), indicating that NEXMIF overexpression does not disturb physical development. Behavioral tests were conducted during the adolescent period between P30 to P70, and the brains of injected mice were collected for various biochemical analyses at either P40 or P70 (Figure 1F). By RT-qPCR, we found that cortical tissue from NEX^OX^ mice at P40 and P70 showed significant expression of human *NEXMIF* mRNA relative to CTRL mice, confirming successful integration and expression of LV-NEXMIF in the injected mice (Figure 1G). Consistently, western blotting for NEXMIF revealed significantly increased NEXMIF expression in the medial prefrontal cortex (mPFC) of NEX^OX^ mice at both P40 and P70 timepoints (Figures 1H,I). Brain slice images also revealed increased NEXMIF immunosignal in the cortex and hippocampal CA1 (Supplementary Figures 3, 4), and western blotting of P40 hippocampal tissue consistently showed increased NEXMIF expression in NEX^OX^ mice (Supplementary Figure 5). Taken together, these findings validate the efficacy of LV-NEXMIF in increasing NEXMIF expression in the mouse brain.

### NEXMIF overexpression alters communication in neonatal mice

**Figure 2:**
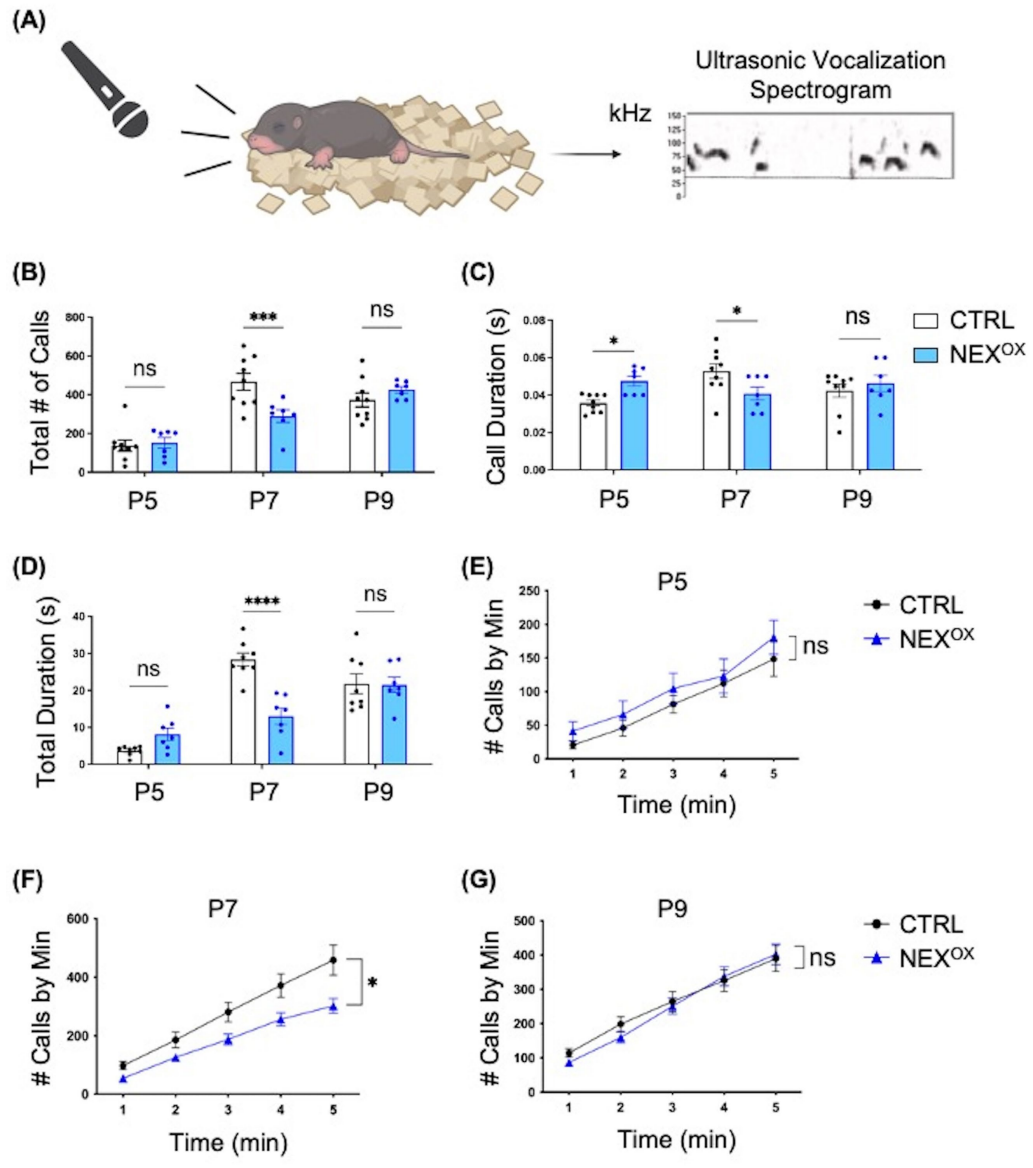
NEXMIF overexpression alters communication in neonatal mice. **(A)** Left: Schematic of the ultrasonic vocalizations (USV) test. Mouse pups aged postnatal (P) day 5, 7 or 9 are isolated from the dam and singly placed in a recording chamber for 5 min. Right: Representative spectrogram of vocalizations from P5 recordings of CTRL mice, 0.4 s in length. Dashed line depicts the 30 kHz frequency threshold for eliminating noise. **(B)** Quantification of the total number of calls at each developmental time point showed a decrease in the number of calls made by NEX^OX^ pups compared with CTRL pups at P7 (CTRL: *n* = 9, NEX^OX^: *n* = 7). **(C)** Quantification of the mean duration of calls emitted at each developmental time point indicated an increase in mean duration at P5 and a decrease at P7 in NEX^OX^ pups compared to CTRL, but no difference at P9. **(D)** Quantification of the total duration of calls emitted at each developmental time point revealed a trend toward an increase in total duration at P5 and a decrease at P7 in NEX^OX^ pups compared to CTRL, but no difference at P9. **(E–G)** Quantification of the average number of calls emitted per minute at each developmental time point. NEX^OX^ pups showed a reduced number of calls during all 5 min compared to CTRL pups at P7 **(F)**, with no differences at P5 **(E)** and P9 **(G)**. Data are represented as average ± SEM. A two-way ANOVA with Tukey’s multiple comparisons test **(B–D)** or a simple linear regression with equal slopes test were used (E-G). *p < 0.05; ***p < 0.001, ****p < 0.0001; ns, Not significant. Figure 2A created with Biorender.com.

One of the three hallmarks of ASD is challenges with social communication (CDC, 2024). We have previously demonstrated that *Nexmif* knockout (KO) male mice show impairments in communication via ultrasonic vocalizations (USVs) at neonatal ages (Gilbert et al., 2020). To determine whether postnatal NEXMIF overexpression disrupts social communication behavior in mice, CTRL and NEX^OX^ mouse pups (ages P5, P7, and P9) were singly placed into a recording chamber for 5 min and their USVs were recorded and analyzed (Figure 2A). Quantification of the total number of calls at each developmental time point showed a decrease in the number of calls made by NEX^OX^ pups compared with CTRL pups at P7, with no alterations in the total number of calls at P5 or P9 (Figure 2B). Quantification of the mean duration of calls emitted indicated an increase in the mean duration at P5, and a decrease at P7, in NEX^OX^ pups compared to CTRL, but no difference at P9 (Figure 2C). Consistently, quantification of the total duration of calls emitted revealed a trend toward an increase in total duration at P5, and a decrease at P7, in NEX^OX^ pups compared to CTRL, but no difference at P9 (Figure 2D). Lastly, quantification of the average number of calls emitted per minute revealed that NEX^OX^ pups showed a reduced number of calls throughout all 5 min compared to CTRL pups at P7 (Figure 2F), with no differences at P5 (Figure 2E) or at P9 (Figure 2G). These findings suggest that NEXMIF overexpression in mice is associated with impaired social communication at P7, as demonstrated by reduced call number and call duration.

### NEXMIF overexpression impairs novel object discrimination in adolescent mice

**Figure 3:**
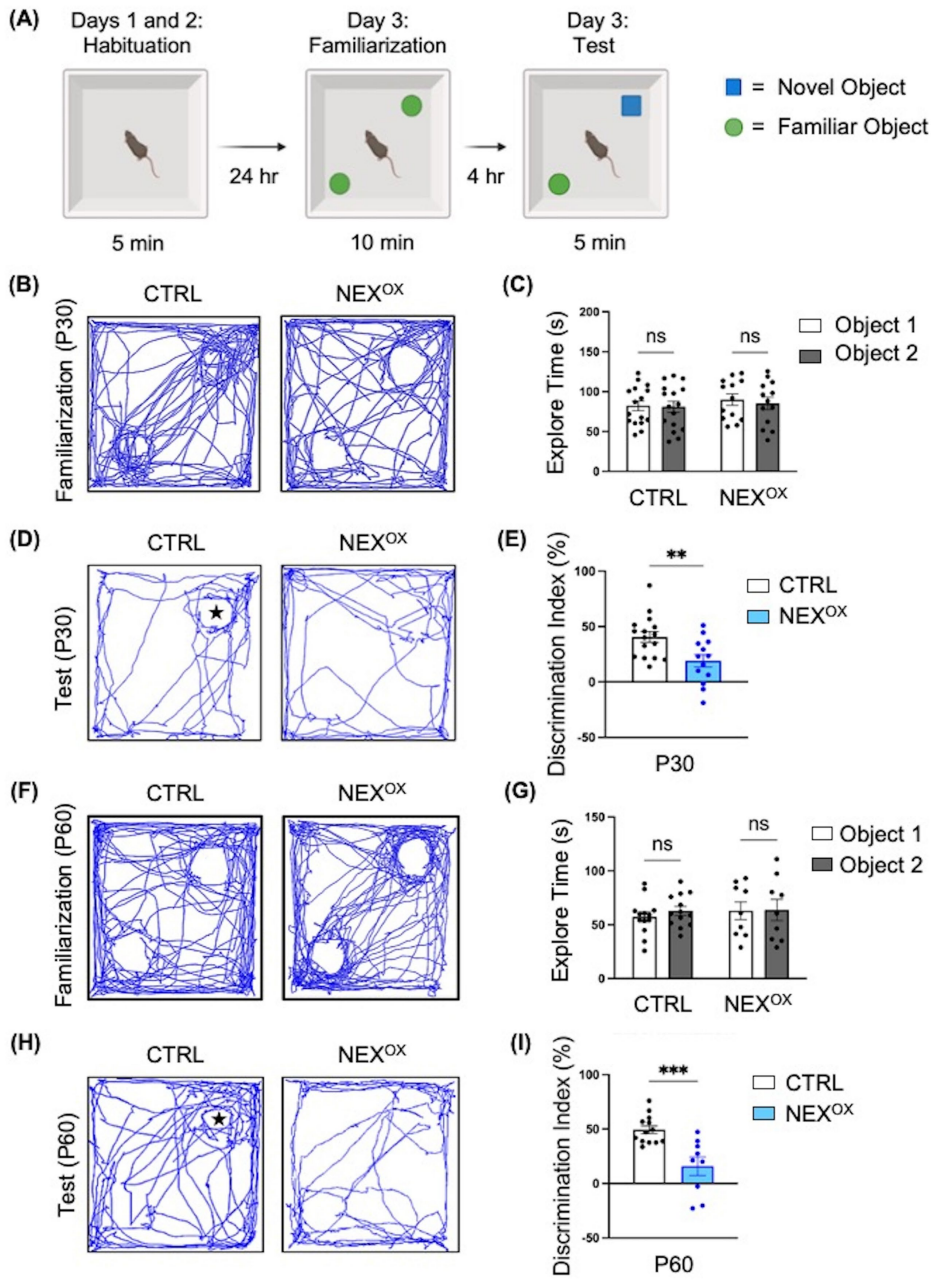
NEXMIF overexpression impairs novel object discrimination in adolescent mice. **(A)** Paradigm for the Novel Object Recognition Test. **(B)** Traces of mouse track paths during the familiarization phase for P30 CTRL and NEX^OX^ mice. **(C)** Quantification of the exploration time during familiarization revealed no preference for either of the identical objects within each group of injected P30 mice (CTRL: *n* = 16, NEX^OX^: *n* = 13). **(D)** Traces of mouse track paths during the test phase for P30 CTRL and NEX^OX^ mice. The star indicates the location of the novel object. **(E)** Quantification of the novelty Discrimination Index (DI). P30 NEXOX mice showed impaired discrimination for the novel object, relative to CTRL. DI: [(Time at Novel – Time at Familiar) / (Time at Novel + Time at Familiar) *100]. **(F)** Traces of mouse track paths during the familiarization phase for P60 CTRL and NEX^OX^ mice. **(G)** Quantification of the exploration time during familiarization revealed no preference for either of the identical objects within each group of injected P60 mice (CTRL: *n* = 13, NEX^OX^: *n* = 9). **(H)** Traces of mouse track paths during the test phase for P60 CTRL and NEX^OX^ mice. **(I)** Quantification of the novelty DI. P60 NEX^OX^ mice also showed significantly impaired discrimination for the novel object, relative to CTRL. Data are represented as average ± SEM. Two-way ANOVA with Tukey’s multiple comparisons test **(C,G)** or a student’s two-tailed *t* test **(E,I)**. **p < 0.01; ***p < 0.001; ns, Not significant. Figure 3A created with Biorender.com.

*Nexmif* KO male and HET female mice display impaired learning and memory in the Barnes maze and fear conditioning tests (Gilbert et al., 2020; O’Connor et al., 2024). To determine whether NEXMIF overexpression is associated with memory deficits, we conducted the Novel Object Recognition (NOR) test to assess deficits in short-term recognition memory in adolescent NEX^OX^ mice. During the NOR test, mice are first habituated to the NOR arena, familiarized to two identical objects, and then tasked with discriminating a novel object from the familiar object 4 h later (Figure 3A; Leger et al., 2013; Lueptow, 2017; Qiao et al., 2022; Tian et al., 2024). The amount of time spent sniffing and interacting with each object (i.e., the exploration time) was recorded and used to calculate the novelty discrimination index as a measure of short-term recognition memory. During the familiarization phase, CTRL and NEX^OX^ mice at both P30 and P60 timepoints spent a similar amount of time on average exploring each object, indicating no bias for either of the familiar objects (Figures 3B,C,F,G). During the test phase, calculation of the novelty discrimination index revealed that while the CTRL mice were able to successfully and strongly discriminate the novel object from the familiar object, the NEX^OX^ mice showed significantly reduced discrimination for the novel object, indicating a reduced ability to recall the memory of the familiar object at both P30 and P60 timepoints (Figures 3D,E,H,I). These results suggest that postnatal NEXMIF overexpression can lead to the development of short-term memory deficits in mice and further implicates the role of NEXMIF in cognitive function.

### NEXMIF overexpression impairs social novelty preference in adolescent mice

**Figure 4:**
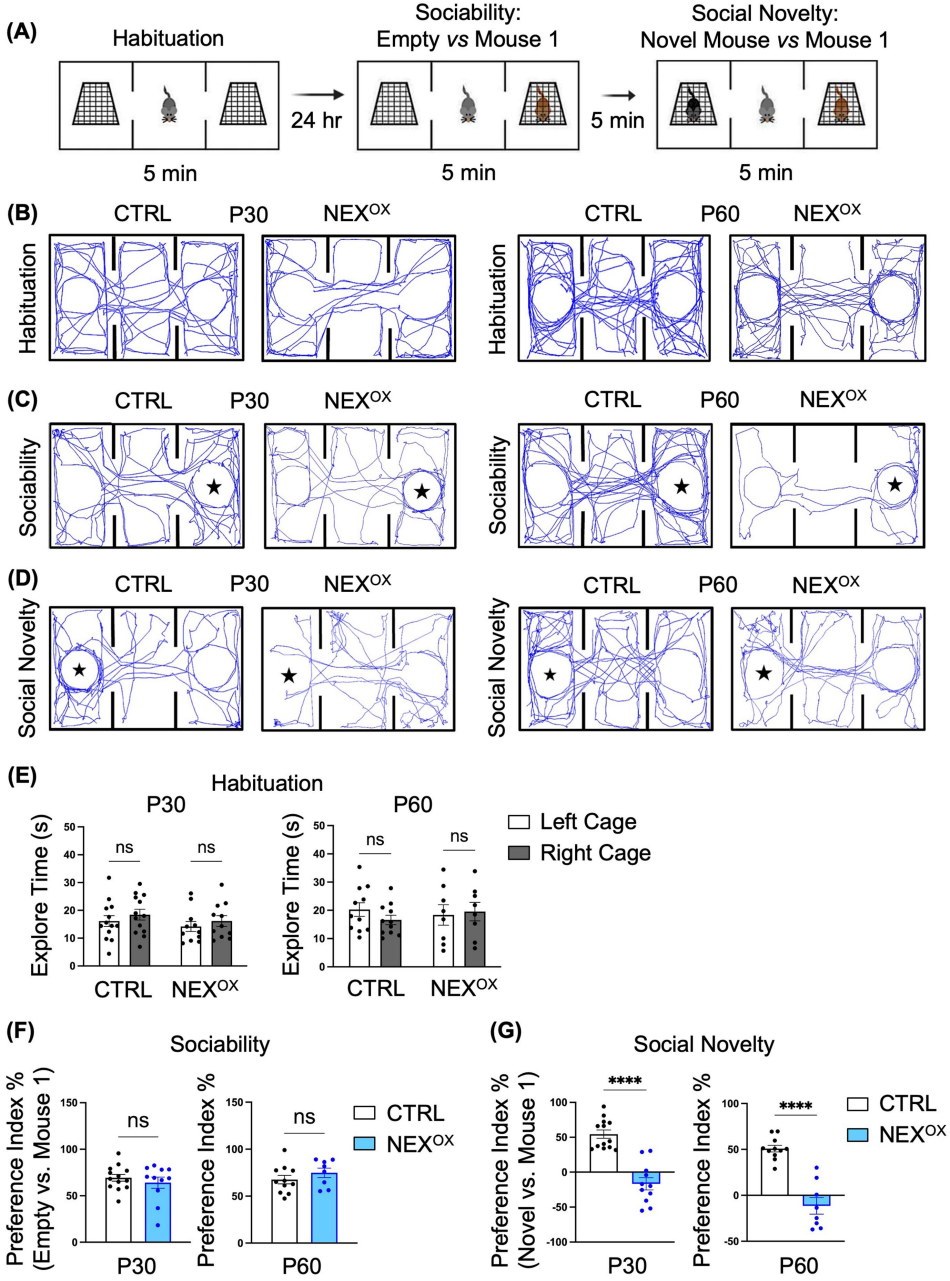
NEXMIF overexpression impairs social novelty preference in adolescent mice. **(A)** Paradigm for the Three-Chamber Social Test. **(B)** Traces of mouse track paths on the first day of habituation for P30 (left) and P60 (right) CTRL and NEX^OX^ mice. **(C)** Traces of mouse track paths in the sociability test for P30 (left) and P60 (right) CTRL and NEX^OX^ mice. The star indicates the location of Mouse 1. **(D)** Traces of mouse track paths in the social novelty test for P30 (left) and P60 (right) CTRL and NEX^OX^ mice. The star indicates the location of the Novel Mouse. **(E)** Quantification of the exploration time during habituation revealed no preference for either side chamber among CTRL and NEX^OX^ mice at P30 (left graph, *n* = 13 and *n* = 11) and P60 (right graph, *n* = 11 and *n* = 8). **(F)** In the sociability test, NEX^OX^ mice showed intact social preference for an unfamiliar mouse at both P30 and P60 timepoints, relative to CTRL mice. **(G)** In the social novelty test, NEX^OX^ mice showed no social preference for the novel mouse at both P30 and P60 timepoints, relative to CTRL mice. Data are represented as average ± SEM. A two-way ANOVA with Tukey’s multiple comparisons test **(E)** or a student’s two-tailed *t* test **(F,G)** were used. ****p < 0.0001. ns, Not significant.

We then performed the Three-Chamber Social test (3CST), which is a well-known social behavior task commonly used to assess mouse sociability and interest in social novelty (Gilbert et al., 2020; O’Connor et al., 2024; Tian et al., 2024; Qiao et al., 2022; Tian et al., 2024; Crawley, 2007; Gardner et al., 2024; Huo et al., 2022; Moy et al., 2009). We previously demonstrated that *Nexmif* KO male and HET female mice show impairments in sociability, accompanied by a lack of interest in social novelty (Gilbert et al., 2020; O’Connor et al., 2024). To examine the effects of NEXMIF overexpression on social behavior, P30 and P60 CTRL and NEX^OX^ mice were subjected to the 3CST. The 3CST is divided into three parts: (1) habituation, during which mice are habituated to the three-chamber apparatus to test for bias toward either of the side chambers containing empty cages; (2) sociability, during which preference for exploring an empty chamber versus a stranger mouse (mouse 1) is measured; and (3) social novelty, during which preference for exploring a familiar mouse (mouse 1) versus a novel mouse is measured (Figure 4A). The amount of time spent sniffing and interacting with each empty cage, mouse 1, and the novel mouse (i.e., the exploration time) was recorded and used to calculate the sociability and social novelty preference indices. During the habituation phase, we found that CTRL and NEX^OX^ mice spent a similar amount of time on average exploring both empty cages, indicating that the mice showed no bias toward exploring either the left or right chambers at both P30 and P60 timepoints (Figures 4B,E). Calculation of the sociability preference index revealed that both P30 and P60 NEX^OX^ mice showed normal preference for exploring the stranger mouse (mouse 1) over the empty cage, like that of CTRL mice, indicating that NEXMIF overexpression does not alter sociability behavior in adolescent mice (Figures 4C,F). However, analysis of the social novelty preference index revealed that while CTRL mice showed a strong preference for exploring the novel mouse over mouse 1 (the now familiar mouse), both P30 and P60 NEX^OX^ mice showed a significantly increased preference for the familiar mouse 1 over the novel mouse, indicating a lack of preference for social novelty (Figures 4D,G). These results demonstrate that while NEXMIF overexpression does not alter sociability behavior, it strongly attenuates preference for social novelty in mice.

### NEXMIF overexpression is associated with hyperactivity and anxiety in mice

**Figure 5:**
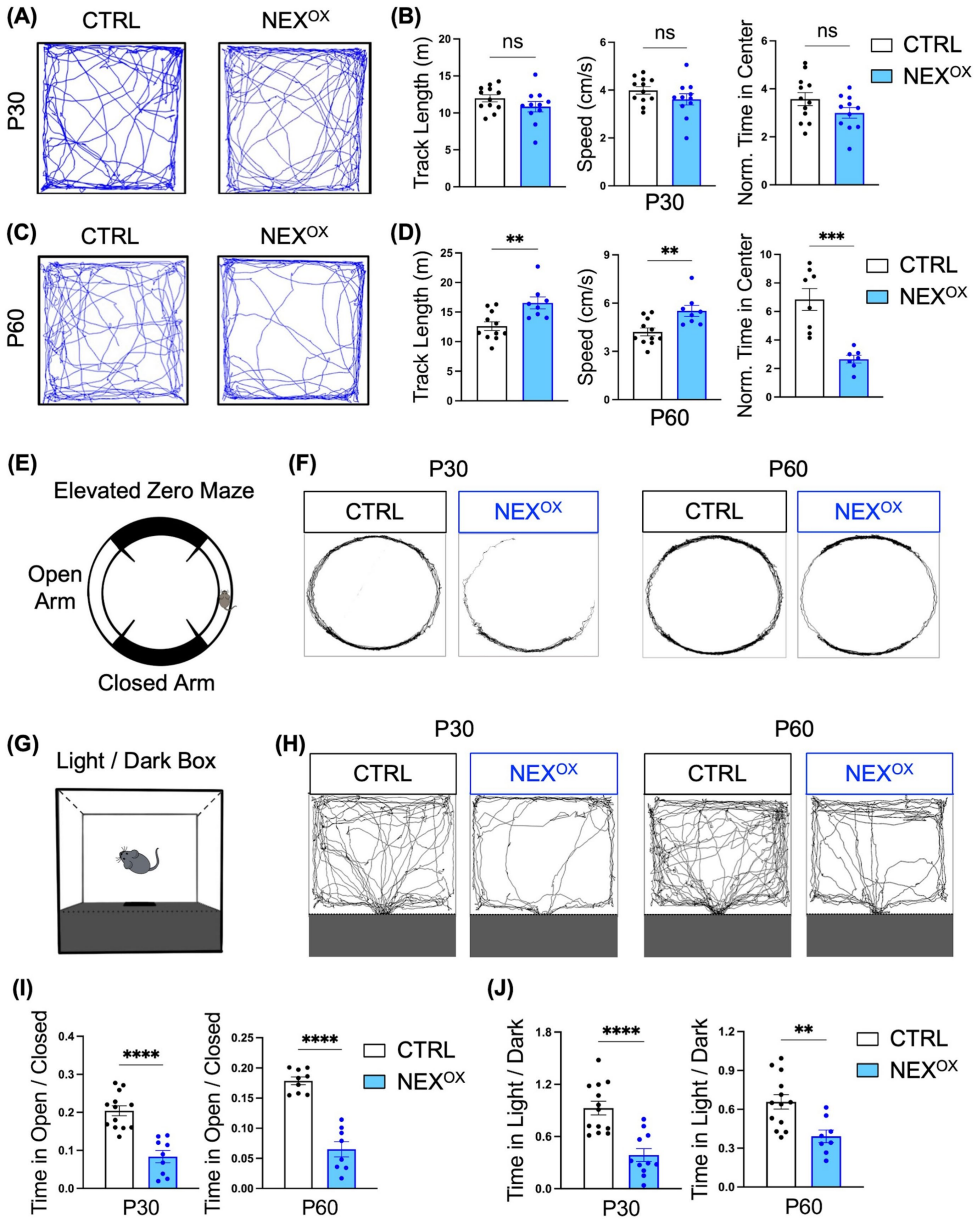
NEXMIF overexpression is associated with hyperactivity and anxiety in adolescent mice. **(A)** Representative tracings of P30 CTRL (*n* = 12) and NEX^OX^ (*n* = 11) mouse track paths during the Open Field test. **(B)** Quantification of the total distance traveled (left graph), the average speed (middle graph), and the normalized time spent in the center of the arena (right graph) of P30 CTRL and NEX^OX^ mice. Time in center is normalized to the total distance traveled for each mouse. **(C)** Representative tracings of P60 CTRL (*n* = 11) and NEX^OX^ (*n* = 8) mouse track paths during the Open Field test. **(D)** Quantification of the total distance traveled (left graph), the average speed (middle graph), and the normalized time spent in the center of the arena (right graph) of P60 CTRL and NEX^OX^ mice. Time in center is normalized to the total distance traveled for each mouse. **(E)** Illustration of the Elevated Zero Maze (EZM) apparatus. **(F)** Traces of mouse track paths during the EZM test for CTRL and NEX^OX^ mice at P30 (left) and P60 (right) timepoints. **(G)** Illustration of the Light/Dark box (LDB). **(H)** Traces of mouse track paths in the LDB test for CTRL and NEX^OX^ mice at P30 (left) and P60 (right) timepoints. **(I)** Quantification of the EZM test revealed that both P30 and P60 NEX^OX^ mice (*n* = 9 and *n* = 8) spent significantly less time in the open arms than CTRL mice (P30: *n* = 13; P60: *n* = 13). **(J)** Consistently, quantification of the LDB assay revealed that both P30 and P60 NEX^OX^ (*n* = 11 and *n* = 8) mice also spent significantly less time in the light compartment relative to CTRL mice (P30: *n* = 13; P60: *n* = 13). Data are represented as average ± SEM. A student’s two-tailed *t* test **(B,D,I,J)** was used. **p < 0.01; ***p < 0.001; ****p < 0.0001. ns, Not significant.

Because ASD is often co-morbid with attention-deficit/hyperactivity disorder (ADHD) and anxiety disorder (Al Ghamdi and AlMusailhi, 2024; Guerrera et al., 2022; Hours et al., 2022; van Steensel et al., 2011), we next sought to determine the effects of NEXMIF overexpression on hyperactivity and anxiety-like phenotypes in NEX^OX^ mice. The Open Field (OF) assay is a commonly used test to measure locomotor activity (distance traveled and speed) and anxiety-related emotional behaviors (Carola et al., 2002; Seibenhener and Wooten, 2015). Our previous study revealed that *Nexmif* KO male and HET female mice travel longer distances at higher speeds and spend significantly less time in the center of the OF arena when compared to controls, phenotypes commonly associated with hyperactivity and increased anxiety, respectively (Gilbert et al., 2020; O’Connor et al., 2024). Using the OF assay, we found that P30 CTRL and NEX^OX^ mice showed no differences in total distance traveled or average speed, while the time spent in the center of the arena was slightly reduced in NEX^OX^ mice (Figures 5A,B). However, by the age of P60, NEX^OX^ mice displayed a significant increase in total distance traveled and average speed, accompanied by a stark reduction in time spent in the center of the arena when compared to CTRL mice (Figures 5C,D), indicating the presence of hyperactivity and anxiety-like behavior.

To further determine the extent of anxiety-driven behavior in NEX^OX^ mice, we employed the elevated zero maze (EZM) test and light dark box (LDB) test given the established sensitivity of these tests for measuring innate anxiety (Bourin and Hascoët, 2003; Kulkarni et al., 2007). The EZM is a circular track version of the standard elevated plus maze (Figure 5E), in which the amount of time spent in the open arms relative to the total distance traveled is measured, while the LDB test measures the amount of time spent in the light portion of the arena relative to the dark portion (Figure 5G). We found that while CTRL mice spent a normal amount of time exploring both the open and closed arms of the EZM, both P30 and P60 NEX^OX^ mice spent a strikingly reduced amount of time exploring the open arms (Figures 5F,I). We found a similar presence of anxiety-like behavior during the LDB assay, in which both P30 and P60 NEX^OX^ mice explored the light compartment significantly less than CTRL mice (Figures 5H,J). Taken together, these results demonstrate that postnatal NEXMIF overexpression can lead to the presence of hyperactivity and anxiety-like behaviors in mice.

### NEXMIF overexpression is associated with repetitive and restrictive behaviors and altered pain response

**Figure 6:**
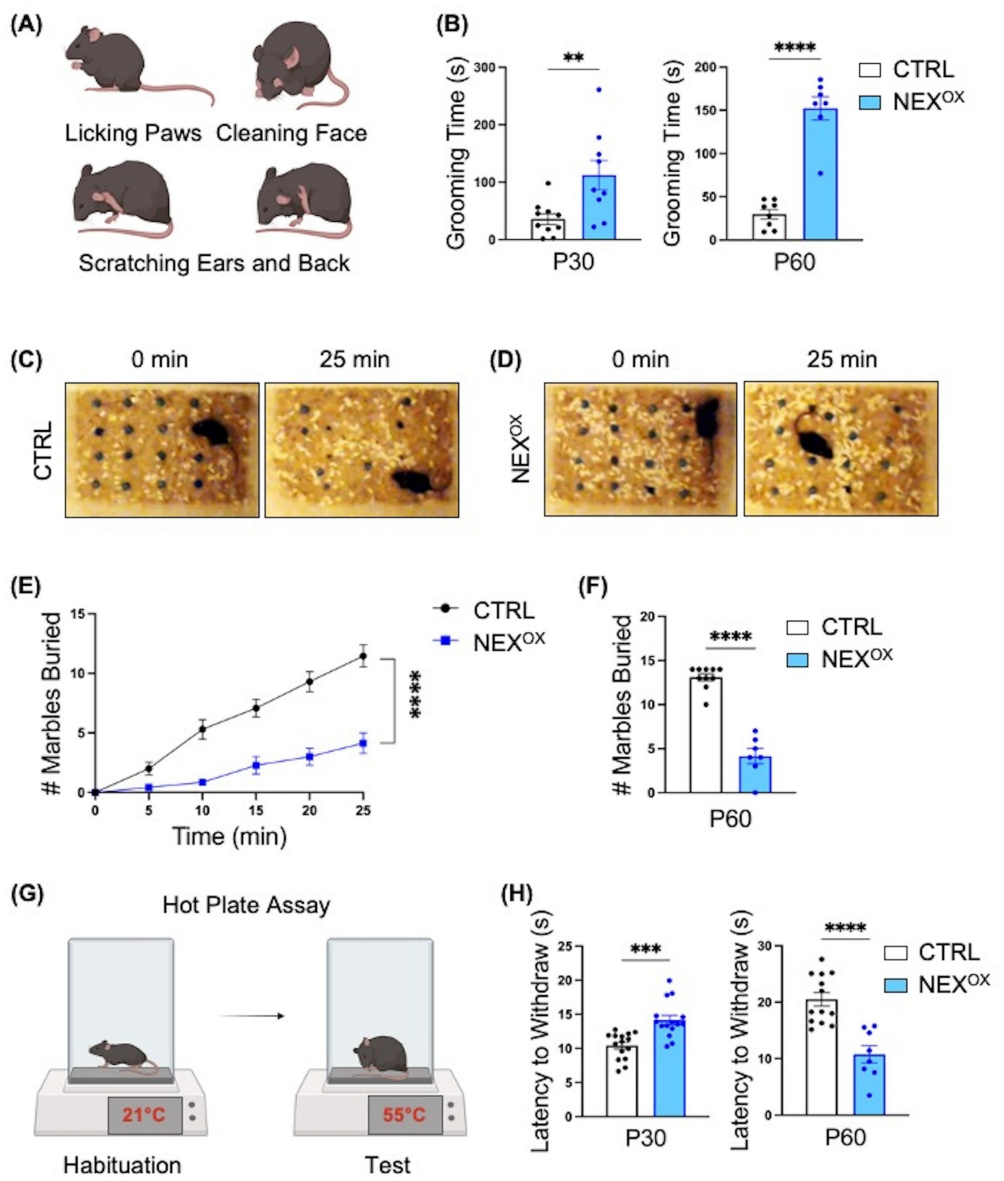
NEXMIF overexpression is associated with repetitive and restrictive behaviors and altered pain response. **(A)** Schematic of grooming behaviors: during the 10-min grooming assay, mice were singly placed in their home cage and the amount of time spent grooming (paw licking, face cleaning, and back/ear scratching) was recorded. **(B)** Relative to CTRL mice, NEX^OX^ mice spent an increased amount of time grooming on average at both the P30 (left graph, *n* = 10 and *n* = 9) and P60 (right graph, *n* = 8 and *n* = 7) timepoints. **(C,D)** Representative images of CTRL **(C)** and NEX^OX^ **(D)** mice at the start (0 min) and end (25 min) of the Marble Burying test (P60 only). **(E)** P60 NEX^OX^ mice demonstrated restrictive behavior by burying significantly less marbles every 5 min relative to CTRL mice. **(F)** Quantification of the average number of marbles buried within 25 min. **(G)** Schematic of Hot Plate Assay. **(H)** Quantification of latency to withdraw in P30 (*n* = 15) and P60 (*n* = 8) mice. Data are represented as average ± SEM. A student’s two-tailed *t* test **(B,F,H)** or a simple linear regression with equal slopes test **(E)** were used. **p < 0.01; ***p < 0.001; ****p < 0.0001. Figures 6A,G created with Biorender.com.

We also examined the effects of NEXMIF overexpression on repetitive/restrictive behaviors, which are one of the hallmark features of ASD in humans (CDC, 2024; Maenner, 2023). Indeed, we previously found that *Nexmif* KO male and HET female mice show severe repetitive overgrooming behaviors to the point of fur loss at the site of grooming, which has been linked to anxiety (Gilbert et al., 2020; O’Connor et al., 2024; Kalueff et al., 2016). Furthermore, the Marble Burying test is an assay used to examine restrictive behavior and has been shown to depend on an animal’s interest in the external environment (Greco et al., 2013; Thomas et al., 2009). Marble burying behavior was significantly reduced in *Nexmif* KO and HET mice, indicating that *Nexmif* loss in mice is associated with the presence of restriction/lack of interest. We therefore wanted to examine whether NEXMIF overexpression similarly leads to the development of repetitive and restrictive ASD-like behaviors. During the grooming assay, mice were video recorded in their home cage for 10 min, and the total amount of time spent grooming (Figure 6A: paw lick, face cleaning, and ear or back scratching) was measured. Both P30 and P60 NEX^OX^ mice spent a significantly longer amount of time grooming than CTRL mice (Figure 6B), with a striking ~175% increase in grooming time at P30 and ~500% increase at P60, indicating that NEXMIF overexpression is associated with repetitive overgrooming behavior. We then used the Marble Burying task to examine the presence of restrictive behavior. Mice were singly placed in a cage of fresh bedding with a 4×4 grid of marbles and recorded for 25 min (Figures 6C,D). Like *Nexmif* KO male and HET female mice, we found that P60 NEX^OX^ mice buried significantly fewer marbles than CTRL mice at each 5-min interval (Figure 6E) and on average throughout the entire 25-min observation period (Figure 6F). These findings suggest that NEXMIF overexpression can increase repetitive behaviors and extensively reduce a mouse’ interest in the external environment, two phenotypes consistent with human ASD.

Sensory abnormalities, such as both hyper- and hypo-responsiveness to particular stimuli, are also commonly reported in individuals with ASD (Balasco et al., 2019; Marco et al., 2011; Rogers and Ozonoff, 2005). In mice, the use of thermal noxious stimuli has been the basis for several well-known tests designed to assess analgesic reactions to pain-relieving compounds (Barrot, 2012; Deuis et al., 2017). The Hot Plate assay is one such test in which a mouse is placed on a ceramic surface heated to 55°C, and the amount of time that passes until the mouse reacts by paw-licking and/or jumping is recorded as the “latency to withdraw” (Figure 6G; Qiao et al., 2022; Tian et al., 2024; Duman et al., 2006; Menéndez et al., 2002). Indeed, knockout of the ASD gene *Shank2* has been associated with an increased latency time (hypo-responsiveness) in mice, while *Cntnap2* knockout mice show a reduced latency time (hyper-responsiveness), indicating a gene-specific effect on thermal nociception in ASD mouse models (Dawes et al., 2018; Ko et al., 2016). Here, we found that postnatal NEXMIF overexpression led to an age-dependent effect on thermal nociception in mice. At P30, NEX^OX^ mice demonstrated a longer latency to withdraw relative to CTRL mice; however, at P60, NEX^OX^ mice became more sensitive to the noxious heat stimulus as demonstrated by a significantly reduced latency to withdraw relative to CTRL mice (Figure 6H). These findings suggest that although normal somatosensory processing needs intact peripheral detection and spinal cord relay to the cortex, overexpression of NEXMIF in the brain is sufficient to alter sensory processing in mice.

### NEXMIF overexpression induces dendritic branching in primary neurons

**Figure 7:**
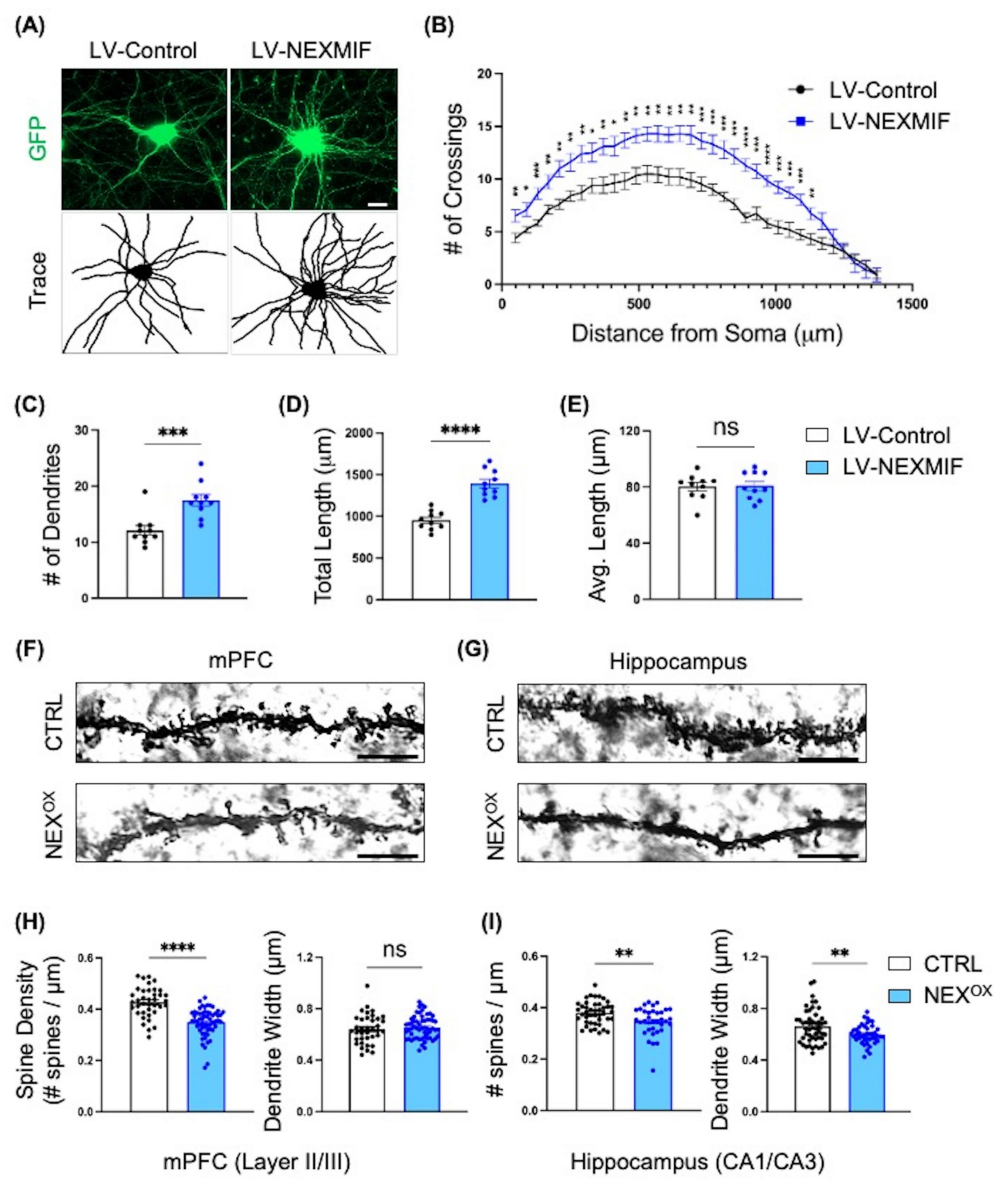
NEXMIF overexpression induces dendritic branching in vitro and impairs spine morphology in adolescent mice. **(A)** Cultured primary cortical neurons were infected with LV-GFP together with either LV-Control or LV-NEXMIF at DIV 3 and fixed for imaging at DIV 10. Top row: GFP; Bottom row: tracing of dendrites. Scale bar = 10 μm. **(B)** Sholl analysis of the neurons from **(A)** showed that NEXMIF overexpression significantly increased the number of dendritic crossings throughout the length of the neuron, relative to control. **(C–E)** Structural analysis of the neurons from A revealed that NEXMIF overexpression significantly increased the average number of dendrites **(C)** and the total dendritic length **(D)**, but did not induce changes in the average dendritic length **(E)**. **(F,G)** Representative Golgi stain images of basolateral dendritic spines in the mPFC (Layer 2/3, **F**) and Hippocampus (CA1/CA3, **G**) from P40 CTRL (top row) and NEX^OX^ (bottom row) mice. Scale bar = 4 μm. **(H,I)** Quantification of spine density (left panel) and dendrite width (right panel) revealed that while NEXMIF overexpression led to decreased spine density and unaltered dendrite width in layer 2/3 cortical neurons **(H)**, both spine density and dendrite width were significantly reduced in the hippocampal CA1/CA3 neurons of NEX^OX^ mice **(I)**. *N* = 40–60 dendrites from 20 to 25 neurons per condition. Data are represented as average ± SEM. Two-tailed student’s *t* test **(C–E,H,I)** or an unpaired Multiple *t* test and Area Under the Curve analysis **(B)**. *p < 0.05; **p < 0.01; ***p < 0.001; ****p < 0.0001. ns, Not significant.

After confirming the effects of NEXMIF overexpression on mouse behavior, we then wanted to examine potential defects at the cellular level. We and others have previously shown that loss of *Nexmif* is associated with stunted neurite outgrowth and branching and impaired spine morphology in cultured neurons and in the mouse brain (Ishikawa et al., 2012; Gilbert et al., 2020; Gilbert and Man, 2016; O’Connor et al., 2024). We thus wanted to determine whether overexpression of NEXMIF is also associated with impaired neuron morphology. To examine the effect of NEXMIF overexpression on dendritic morphology, cultured rat cortical neurons were co-infected with LV-GFP and either LV-Control or LV-NEXMIF at DIV 3, followed by immunostaining for GFP at DIV 10 to visualize dendritic structure (Figure 7A). Strikingly, Sholl analysis revealed a marked increase in the number of dendritic branches in neurons overexpressing NEXMIF (Figure 7B). Compared with LV-Control-infected neurons, we observed a ~ 45% increase in the number of dendrites and a ~ 46% increase in the total dendritic length in LV-NEXMIF-infected neurons (Figures 7C,D). However, NEXMIF overexpression did not alter average dendritic length (Figure 7E). In addition, Golgi images of dendritic branching *in vivo* in the cortex and hippocampus of P40 NEX^OX^ mice display increased dendritic branching patterns relative to CTRL mice (Supplementary Figure 6). These results indicate that NEXMIF overexpression is associated with enhanced dendritic arborization and length, as compared to the severe stunting of dendritic outgrowth and branching following loss of NEXMIF.

### NEXMIF overexpression impairs spine morphology in adolescent mice

We have previously shown that loss of NEXMIF is associated with reduced spine density and immature spine morphology in the hippocampus of *Nexmif* KO male and HET female mice (Gilbert et al., 2020; O’Connor et al., 2024). Given the presence of autistic-like phenotypes and impaired short-term memory in NEX^OX^ mice, we wondered whether such alterations may have resulted from abnormalities in spine formation, such as reduced spine formation, increased spine pruning, or a lack of stabilization. To examine this, Golgi staining was performed on brains harvested from CTRL and NEX^OX^ mice at P40, followed by quantification of basolateral dendritic spine density and average dendritic width in Layer 2/3 of the medial prefrontal cortex (mPFC; Figure 7F) and in the CA1/CA3 subregions of the hippocampus (HPC; Figure 7G). Layer 2/3 cortical neuron spine density was significantly reduced in NEX^OX^ mice, relative to CTRL mice, with no alterations in dendrite width (Figure 7H). NEX^OX^ mice also displayed reduced spine density in CA1/CA3 pyramidal neurons, in addition to attenuated dendrite width relative to CTRL mice (Figure 7I). These findings suggest that NEXMIF overexpression results in reduced spine density in the mPFC and HPC of mice, which likely leads to abnormal synaptic connectivity in the neural circuitry.

### NEXMIF overexpression results in transcriptional dysregulation in the brain

**Figure 8:**
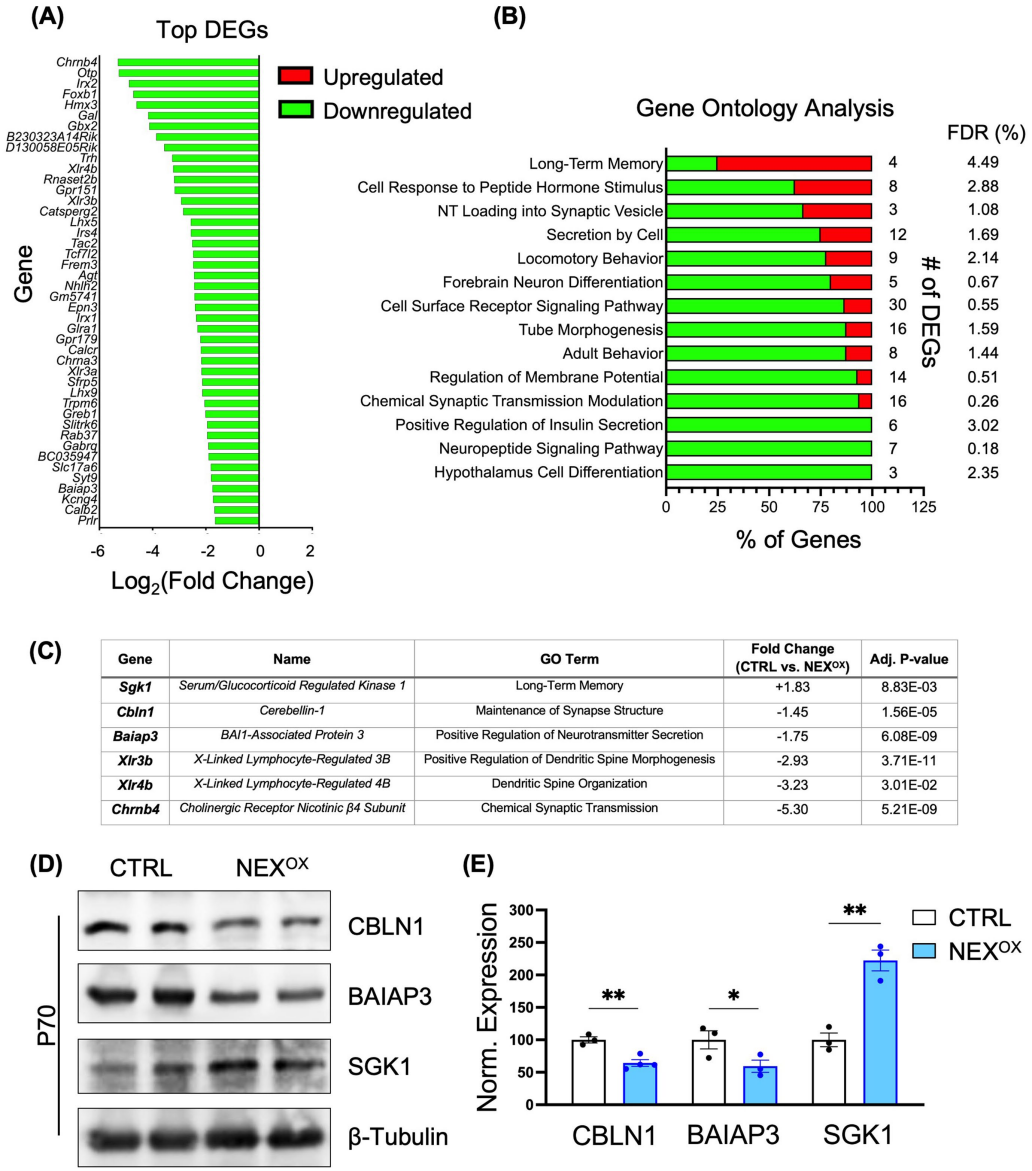
NEXMIF overexpression results in transcriptional dysregulation in the brain. **(A)** RNA sequencing bar chart showing the normalized (Log2) fold changes of the top 44 of 161 total differentially expressed genes (DEGs) in the hippocampus (HPC) of P70 NEX^OX^ mice. In total, 131 DEGs (81%) were downregulated and 30 DEGs (19%) were upregulated by at least a 1.3-fold change in the NEX^OX^ mouse brain, relative to CTRL. **(B)** Gene Ontology (GO) enrichment analysis bar chart depicting the most enriched biological processes. The “# of DEGs” refers to the number of genes from the RNA sequencing dataset within each predefined *Mus musculus* GO biological process. Each bar reflects the percentage of upregulated (red) and/or downregulated (green) DEGs within each GO biological process. The False Discovery Rate (FDR) is a statistical measure of true nulls. For example, an FDR of 3% means that, among all genes considered significant, 3% of these genes are truly null. **(C)** Selected list of top hippocampal DEGs indicating their associated GO term, fold change in expression (NEX^OX^ / CTRL), and adjusted *p* value. **(D)** Western blot showing the protein expression of CBLN1 (top panel), BAIAP3 (middle panel), and SGK1 (bottom panel) in the brain lysates of P70 CTRL and NEX^OX^ mice. **(E)** Quantification of the western blots in **(D)** showed a significant reduction in CBLN1 and BAIAP3 protein in P70 NEX^OX^ brains, in addition to an increase in SGK1 protein, consistent with RNA sequencing data. *N* = 3–4 mice/group. Data are represented as average ± SEM. Two-tailed student’s t test **(E)**: *p < 0.05; **p < 0.01; ***p < 0.001; ****p < 0.0001.

While the biological function for NEXMIF remains unclear, its nuclear localization and molecular structure analysis suggest a role in regulation of gene transcription. To gain more understanding into the potential molecular mechanism(s) of NEXMIF overexpression, we performed RNA sequencing on brain tissue collected from P70 CTRL and NEX^OX^ mice. The analysis of data obtained from hippocampal tissue revealed significant transcriptional dysregulation in the NEX^OX^ mouse: out of a total of 161 differentially expressed genes (DEGs), 131 were downregulated and 30 were upregulated by at least a 1.3-fold change relative to CTRL (Figure 8A). Interestingly, gene ontology (GO) enrichment analysis revealed that the majority of DEGs were strongly involved in biological processes such as neurotransmitter loading into synaptic vesicles, cell differentiation, neuron differentiation, long-term memory, neuropeptide signaling pathway, cellular response to peptide hormone stimuli, locomotory behavior, regulation of membrane potential, cell secretion, modulation of chemical synaptic transmission, and the cell surface receptor signaling pathway (Figure 8B). Within each biological process (long-term memory), the majority of DEGs were significantly downregulated in response to NEXMIF overexpression. Intriguingly, 75% of DEGs identified within the long-term memory GO term were upregulated. One of these genes, Serum/glucocorticoid-regulated kinase 1 (*Sgk1*), was upregulated 1.8-fold in the NEX^OX^ mouse hippocampus (Figure 8C). Previous studies have demonstrated that *Sgk1* upregulation in mice is associated with neurodegeneration, impairments in learning and memory, and anxiety- and depressive-like behaviors (Elahi et al., 2021; Lang et al., 2006; Lang et al., 2010; Liu et al., 2021; Sato et al., 2008), which may contribute to the development of these deficits in NEX^OX^ mice. Several of the top down-regulated DEGs in the NEX^OX^ hippocampus included *Cerebellin1* (*Cbln1*), *Brain-specific Angiogenesis Inhibitor 1-Associated Protein 3 (Baiap3), X-Linked Lymphocyte-Regulated 3B* and *4B (Xlr3b, Xlr4b)*, and *Cholinergic Receptor Nicotinic Beta 4 Subunit* (*Chrnb4*; Figure 8C). Of these top genes, *Chrnb4* and *Baiap3* have been implicated in the regulation of synaptic transmission and synaptic vesicle exocytosis (Duga et al., 2001; Frahm et al., 2011; Sørensen, 2017; Varoqueaux et al., 2002); *Cbln1* is involved in synaptogenesis and synapse maintenance (Hirai et al., 2005; Matsuda et al., 2010; Seigneur and Südhof, 2018; Uemura et al., 2010; Yuzaki, 2011); *Cbln1, Xlr3b* and *Xlr4b* are implicated in dendritic spinogenesis and spine organization (Cubelos et al., 2010; Di Segni et al., 2020; Kusnoor et al., 2010). Moreover, at the behavioral level, loss of *Cbln1* has been associated with impairments in fear memory and spatial learning (Lu et al., 2024; Otsuka et al., 2016), while loss of *Baiap3* is implicated in the development of anxiety and depression (Kim et al., 2021; Wojcik et al., 2013). Further analysis of three of the top DEGs at the protein level confirmed a reduction in CBLN1 and BAIAP3 protein, as well as an increase in SGK1 protein in P70 NEX^OX^ brain lysates (Figures 8D,E). These findings indicate a critical role for NEXMIF in the transcriptional regulation of genes involved in brain development, specifically synaptic transmission, neuronal differentiation, and memory processes, and dysregulation of these processes by NEXMIF overexpression may underlie the observed neuronal impairments and ASD-like behaviors in NEX^OX^ mice.

## Discussion

We developed a human *NEXMIF* lentivirus which was postnatally injected into the brains of newborn C57/BL6 mice to assess the neuronal and behavioral consequences of NEXMIF overexpression. Both male and female NEX^OX^ mice are not prone to seizures, have no fur loss, maintain a healthy bodyweight, and display no motor coordination defects; however, using the USV test to examine communication at postnatal ages, we observed a reduction in the number and duration of ultrasonic vocalizations (USVs) at the P7 time point (6 days post-injection). In our previous studies, we found that loss of NEXMIF is associated with a reduced total number of calls and decreased call duration at P5, P7, and P9 in *Nexmif* KO mice (Gilbert et al., 2020). Here, we found similar communication deficits in NEX^OX^ mice at the P7 time point only, suggesting that while NEXMIF loss has a more profound and robust effect than NEXMIF overexpression on USVs, both cases lead to impaired communication. In normal rodents, USVs typically increase after birth, peak around P6–P8, then decline around P14. It is not yet understood why some rodent models show changes at specific postnatal days (like P7), but not earlier or later. Follow-up studies will test USVs in adult NEX^OX^ mice to examine the presence of specific post-developmental communication impairments. It is important to note that while early postnatal calls can be associated with maternal-seeking behavior, maternal-seeking behavior itself is considered as the first form of social communication that rodent pups display. The atypical social communication observed in *Nexmif* KO and NEX^OX^ pups may lead to diminished maternal care as a result of this reduced ability to interact socially with the mother. Indeed, since USVs appear before the emergence of overt social or cognitive behaviors, they can be indicative of early social circuit dysfunction that manifests behaviorally at adult ages (Portfors and Perkel, 2014; Fischer and Hammerschmidt, 2011; Takahashi et al., 2016; Yoshizaki et al., 2017).

The NEX^OX^ mice also showed a lack of preference for social novelty, increased repetitive overgrooming, and reduced marble burying/restrictive behaviors. These findings are in line with what is observed in our *Nexmif* KO and HET mice (Gilbert et al., 2020; O’Connor et al., 2024), as well as in mice overexpressing other ASD genes, such as *Ube3a* and *Mecp2* (Smith et al., 2011; Tian et al., 2024; Tian et al., 2024; Gardner et al., 2024; Li et al., 2024; Xu et al., 2018; Yu et al., 2020; Zhao et al., 2022), as well as in in knock-out models of these genes (Yu et al., 2020; Born et al., 2017; Judson et al., 2021; Lee et al., 2018; Sonzogni et al., 2018; Xu et al., 2022). Interestingly, the NEX^OX^ mice showed unaltered sociability accompanied by a strong impairment in social novelty preference during the three-chamber social test. Since the mice were familiarized to the apparatus and empty cages prior to the test, and they did not spend more time exploring the “familiar” empty cage relative to the stranger mouse, it is unlikely that their reduced social novelty preference was due to an increased preference for familiarity. However, it is possible that while NEX^OX^ mice are sociable, they may have difficulty distinguishing or showing interest in a new social partner, which may be due to deficits in social recognition memory or reduced motivation for social exploration. Similar findings have been reported in *Shank2*, *Cntnap2*, and *Neuroligin-3* knockout mouse models of ASD (Won et al., 2012; Peñagarikano et al., 2011; Radyushkin et al., 2009). Moreover, the NEX^OX^ mice also showed reduced marble burying behavior. The marble burying test takes advantage of the natural tendency of mice to bury objects. While ASD mouse models often demonstrate altered marble burying behavior (either reduced or increased), the exact biological meaning remains less clear. While increased marble burying is often considered as a sign of greater anxiety or compulsive-like behaviors (Angoa-Pérez et al., 2013; Taylor et al., 2017), a lack of burying behavior in ASD may suggest an overall lack of interest in the environment (Wahl et al., 2021).

In addition to the core ASD traits, we observed the presence of memory impairments and anxiety-like behaviors. We found that NEX^OX^ mice showed impaired short-term memory function in the novel object recognition test, consistent with the short- and long-term spatial and fear memory deficits observed in *Nexmif* KO and HET mice using the Barnes maze and cued/contextual fear conditioning tests (Gilbert et al., 2020; O’Connor et al., 2024). In line with these findings, both the loss and duplication of *Ube3a* and *Mecp2* have also been associated with the development of cognitive impairment and memory deficits in mice (Collins and Neul, 2022; Smith et al., 2011; Tian et al., 2024; Copping et al., 2017; Godavarthi et al., 2012; Jamal et al., 2017; Robinson and Pozzo-Miller, 2019; Su et al., 2023). With respect to anxiety-like behavior, there are several reports of reduced time spent in the center of the Open Field arena, reduced time in the open arms of the elevated zero/plus maze, and in the light compartment of the light/dark box in mouse models of *Ube3a* and *Mecp2* loss and duplication (Tian et al., 2024; Gardner et al., 2024; Yu et al., 2020; Copping et al., 2017; Godavarthi et al., 2012; Jamal et al., 2017; De Filippis et al., 2010; Jiang et al., 2010; Na et al., 2014; Yasui et al., 2014; Yue et al., 2021), and consistently, our *Nexmif* KO and HET mice show reduced time spent in the center.

At the cellular level, we and others have previously shown that NEXMIF loss is associated with decreased dendritic outgrowth, reduced spine density, and immature spine morphology (Van Maldergem et al., 2013; Ishikawa et al., 2012; Magome et al., 2013; Gilbert et al., 2020; Gilbert and Man, 2016; O’Connor et al., 2024). In this study, we found that NEXMIF overexpression increased dendritic arborization in cultured cortical neurons. In addition, Golgi staining revealed a stark reduction in spine density in the medial prefrontal cortex and hippocampus of adolescent NEX^OX^ mice. Given that loss of *Nexmif* is associated with stunted dendritic growth, it is predicted that NEXMIF overdosage may lead to increased dendritic branching and length. However, the unanticipated reduction in spine density could be due to either impaired spinogenesis, overpruning, or an inability of spinogenesis to keep pace with the dendritic expansion. Thus, the overall effect of NEXMIF overexpression on neuronal activity depends on the relative extent of alterations in dendritic and spine growth. In line with our previous and current results, loss of MeCP2 has been shown to lead to decreased dendritic spine density and longer spine necks in layer 5 cortical and pyramidal mouse neurons (Landi et al., 2011; Stuss et al., 2012). Similarly, overexpression of MeCP2 in primary hippocampal neurons is associated with reduced spine density and increased spine lengthening/thinning (Chapleau et al., 2009; Zhou et al., 2006). However, there are mixed findings regarding the effect of MeCP2 overexpression on dendritic arborization: transient overexpression of MeCP2 *in vitro* was found to result in either increased (Jugloff et al., 2005) or decreased (Chapleau et al., 2009; Zhou et al., 2006) dendritic branching in cultured mouse neurons, while *in vivo* MeCP2 overexpression in the motor neurons of Drosophila or in the optic tectum neurons of Xenopus led to reduced arborization (Marshak et al., 2012; Vonhoff et al., 2012). Interestingly, MeCP2 overexpression was more recently found to a have an age-dependent effect on spine density and dendritic arborization *in vivo* in layer 5 pyramidal neurons of Mecp2 Tg1 mice: while spine density and dendritic branching were both initially increased in adolescence (4–8 weeks), they each fell below control levels into adulthood (12–40 weeks) which coincided with the onset of neurobehavioral dysfunction in the Tg1 mice (Jiang et al., 2013).

In line with the current study, a lack of expression or overexpression of another ASD gene, *UBE3A*, leads to alterations in neuronal growth. We previously demonstrated that UBE3A overexpression is associated with attenuated dendritic branching and spine density in cultured rat neurons and *in vivo* in the cortex of *Ube3A* 2X transgenic mice (Gardner et al., 2024; Khatri et al., 2018). Loss of *Ube3a* (*Ube3a*^m−/p+^) in mice has also been associated with reduced dendritic spine density and decreased terminal dendritic branching (Khatri and Man, 2019). Intriguingly, layer 5 pyramidal neurons in the primary visual cortex of *Ube3a*^m−/p+^ mice show increased spine loss during adolescence; however, when raised in the dark, there was no difference in spine density between control and *Ube3a*^m−/p+^ mice, indicating impaired experience-driven spine maintenance as a result of *Ube3a* loss (Kim et al., 2016).

By RNA sequencing, we identified dysregulated genes in the hippocampus of NEX^OX^ mice, with a large majority of the DEGs being downregulated. Many of the downregulated genes are involved in the regulation of neuron differentiation, dendritic spine morphology, synaptogenesis, anxiety-like behavior, and learning and memory function. Similarly, RNA sequencing of *Mecp2* duplication has also demonstrated a majority of downregulated DEGs involved in tissue patterning, development, neuron differentiation and nervous system processes (Bajikar et al., 2024), suggesting that the dysregulation of these ASD genes affects shared cellular processes contributing to the neuronal and behavioral deficits observed in ASD mouse models. In addition, RNA sequencing of pancreatic tissue from P40 CRISPR/Cas9-generated *Nexmif* knockout mice showed downregulation of *H3f3b*, a gene that plays a critical role in the maintenance of genomic stability and in the regulation of insulin-secreting beta cell proliferation (Stekelenburg et al., 2022; Jang et al., 2015; Paul et al., 2016). Interestingly, we observed that 6 DEGs associated with the GO term “Positive Regulation of Insulin Secretion” were significantly downregulated in NEX^OX^ mice, suggesting reduced or impaired beta cell activity (see Supplementary Table 2). It is likely that both the loss and overdosage of NEXMIF may lead to genomic instability in several tissues, particularly in the brain and pancreas, which is consistent with the comorbidity of ASD/ID and non-autoimmune diabetes among several patient case reports (Stekelenburg et al., 2022). Future investigations on transcriptional changes in the prefrontal cortical tissue of NEX^OX^ mice will help to explain the observed deficits in social cognition, repetitive behaviors, and sensory abnormalities (Mohapatra and Wagner, 2023; Gandhi and Lee, 2021).

Alterations in several brain regions, such as the PFC, HPC, amygdala, and anterior cingulate cortex, have been implicated in the development of ASD. Our study included cellular and molecular analyses of the NEX^OX^ HPC given that mutations in the *NEXMIF* gene are strongly associated with ID in humans, and our NEX^OX^ mice showed strong impairments in learning and memory function. There is growing evidence that HPC-dependent memory impairments are not just comorbid, but may be core features in some ASD subtypes. With respect to the more overt ASD phenotypes, at the social behavior level, the CA2 subregion was shown to be critical for recognizing familiar individuals, and mice with CA2 lesions show deficits in social recognition (Hitti and Siegelbaum, 2014). Moreover, the HPC has been linked to contextual coding of social events, social navigation via the generation of social-spatial maps, and modulation of emotional responses during social interaction (Tavares et al., 2015; Meira et al., 2018). At the social communication level, HPC lesions were shown to impair ultrasonic vocalizations and social investigation (Hermans et al., 2024). In addition, the HPC has also been shown to support behavioral flexibility, such that hyperexcitable HPC circuits have been associated with the presence of repetitive behaviors in ASD individuals (Chen et al., 2024; Hashimoto et al., 2021). In line with this, several transcriptomic studies from both mouse and human postmortem tissue have demonstrated ASD-linked gene expression changes in the hippocampus, such as the dysregulation of synaptic genes and pathways related to neuronal development and plasticity (Yoo et al., 2022; Rexrode et al., 2024). Our hippocampal RNA-sequencing dataset reveals hippocampal-specific molecular signatures associated with NEXMIF overexpression. Follow-up studies will compare transcriptomic alterations in HPC vs. PFC tissue.

To date, no other rodent model of NEXMIF duplication syndrome has been created or studied. The advantage of our lentivirus-induced NEXMIF overexpression model is that it produces mice with roughly doubled NEXMIF protein expression, allowing for us to for the first time study the downstream effects of NEXMIF overdosage at the molecular, cellular, and behavioral levels. However, the levels of NEXMIF overexpression across brain regions may not accurately reflect the physiological pattern of the congenital NEXMIF duplication observed in humans. Also, since the viral promoter is ubiquitous, there may have been expression of NEXMIF in cell types in which it is not normally expressed (e.g., glia). Regarding the timing of virus application, postnatal overexpression limits our ability to study the effects of NEXMIF overdosage on embryonic brain development and its contribution to autistic-like phenotypes and other deficits. Transgenic mice overexpressing NEXMIF will be considered in future studies.

## Data Availability

The data supporting the findings of this study are available within the article and its supplementary materials. The full RNA sequencing data can be accessed and downloaded from https://dx.doi.org/10.6084/m9.figshare.29274269.
