# Supplementary material for: NEXMIF overexpression is associated with autism-like behaviors and alterations in dendritic arborization and spine formation in mice
Source: Front Neurosci. 2025 Jun 18;19:1556570. doi: 10.3389/fnins.2025.1556570 (PMC12215126; doi:10.3389/fnins.2025.1556570)
Supplement: Supplementary file 1 [file Image_1.pdf]

Supplementary Figure S1.

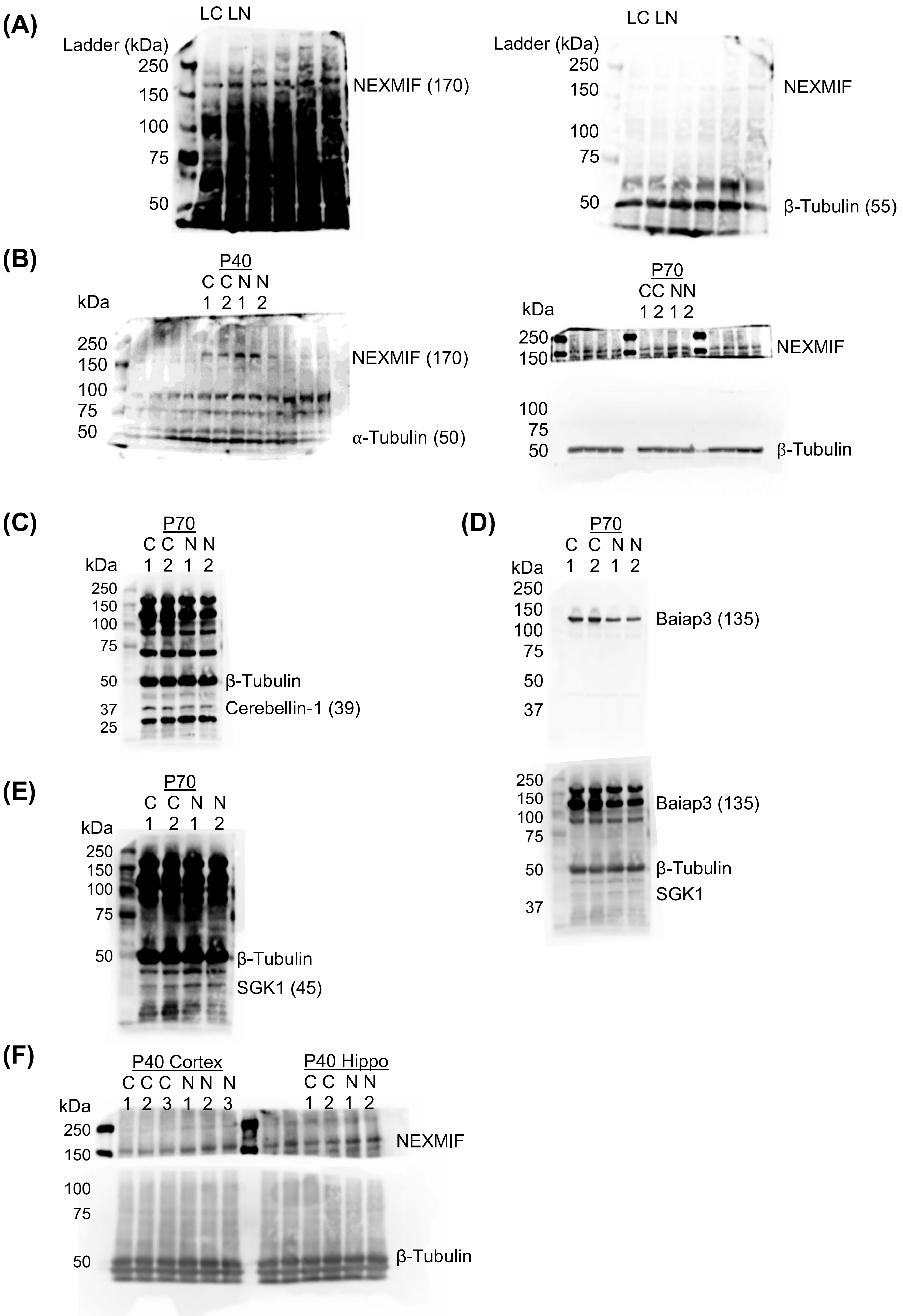

Supplementary Figure S1.

Supplementary Figure S1. Raw Western Blots

(A) Raw western blot for Figure 1B. LC = LV-Control; LN = LV-NEXMIF

(B) Raw western blot for Figure 1H. P40 Blot (left panel) and P70 cut Blots (right panel). C = CTRL; N = NEX^{OX}

(C) Raw western blot for Figure 8D: Cerebellin-1 and β-Tubulin. C = CTRL; N = NEX^{OX}

(D) Raw western blot for Figure 8D: Baiap3 (top) and β-Tubulin (bottom).

(E) Raw western blot for Figure 8D: SGK1 and β-Tubulin.

(C – E: Same blot probed 4 times for the 4 respective antibodies)

(F) Raw western blot for Supplementary Figure 5A-B: NEXMIF and β-Tubulin.
