# Supplementary figures and images for: NEXMIF overexpression is associated with autism-like behaviors and alterations in dendritic arborization and spine formation in mice
Source: Front Neurosci. 2025 Jun 18;19:1556570. doi: 10.3389/fnins.2025.1556570 (PMC12215126; doi:10.3389/fnins.2025.1556570)

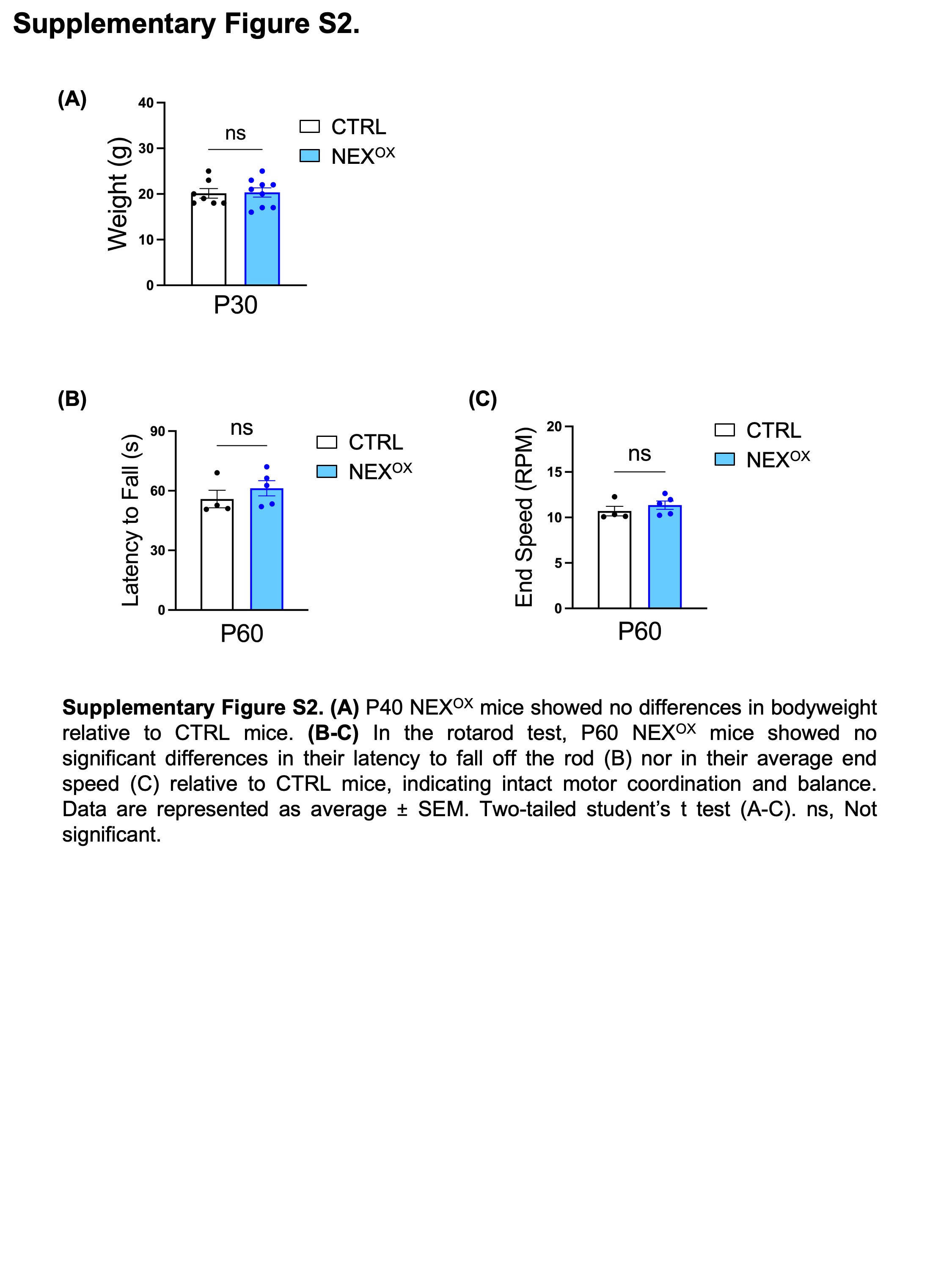

Supplement: Supplementary file 2 [file Image_2.jpeg]

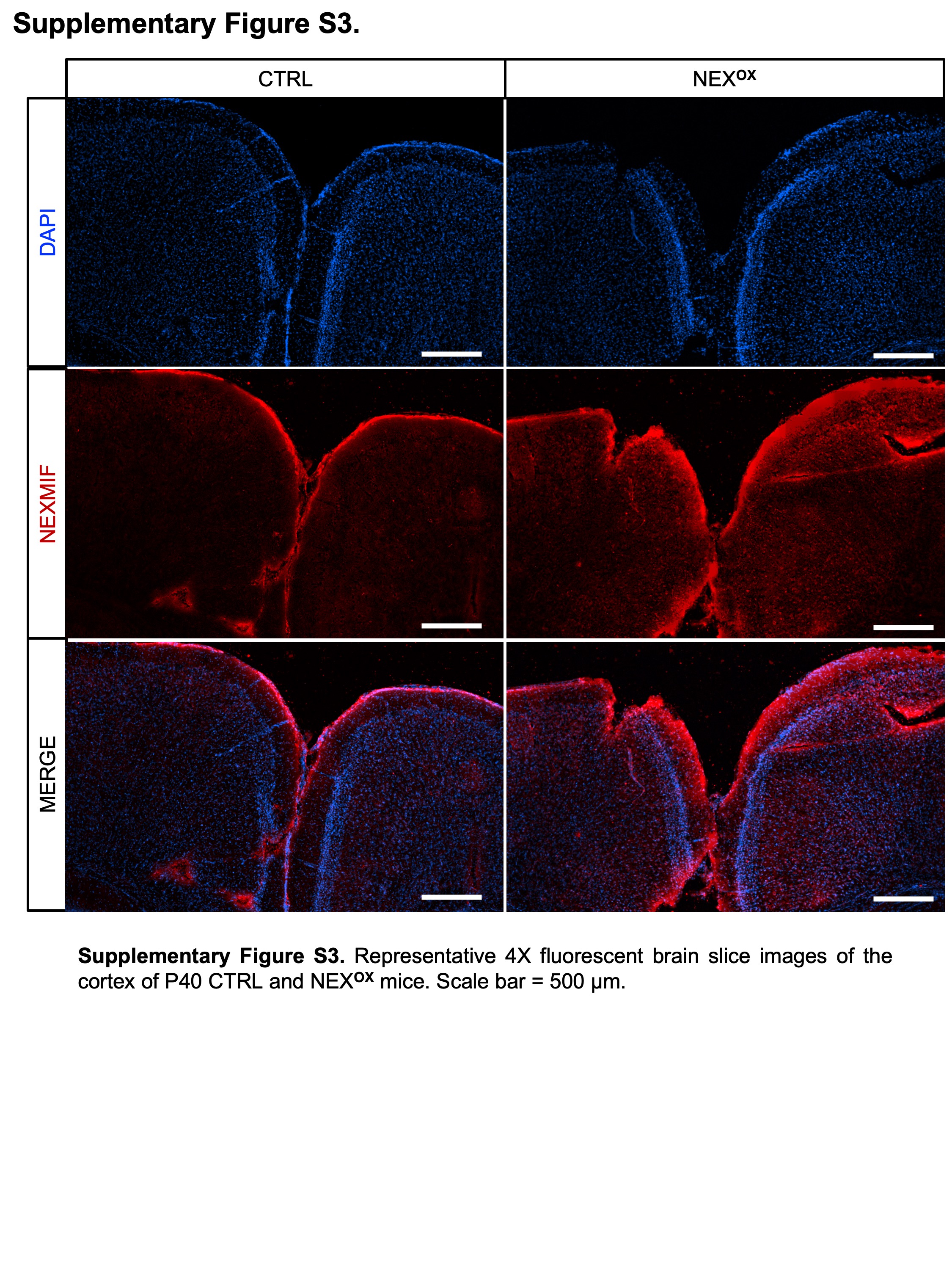

Supplement: Supplementary file 3 [file Image_3.jpeg]

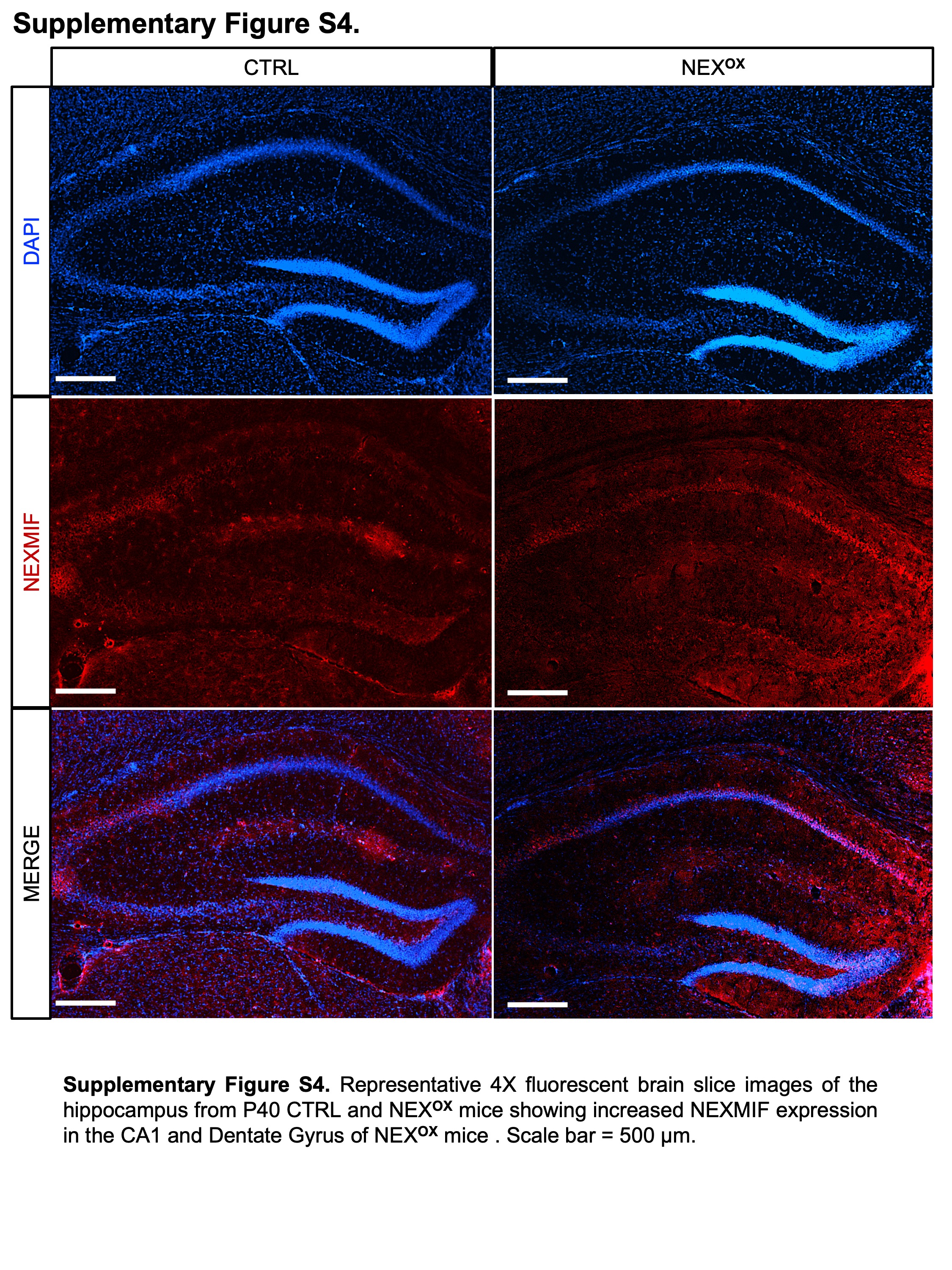

Supplement: Supplementary file 4 [file Image_4.jpeg]

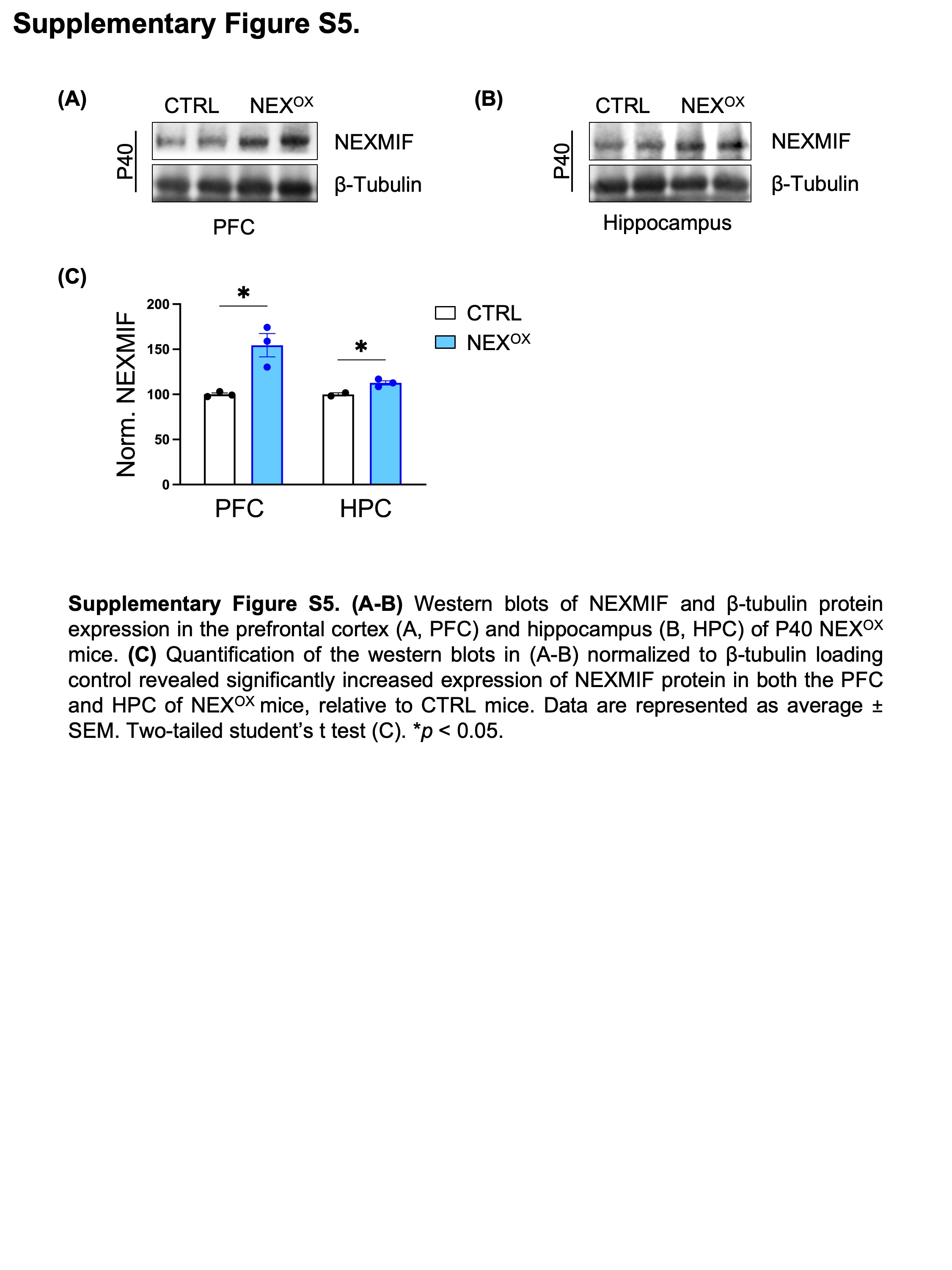

Supplement: Supplementary file 5 [file Image_5.jpeg]

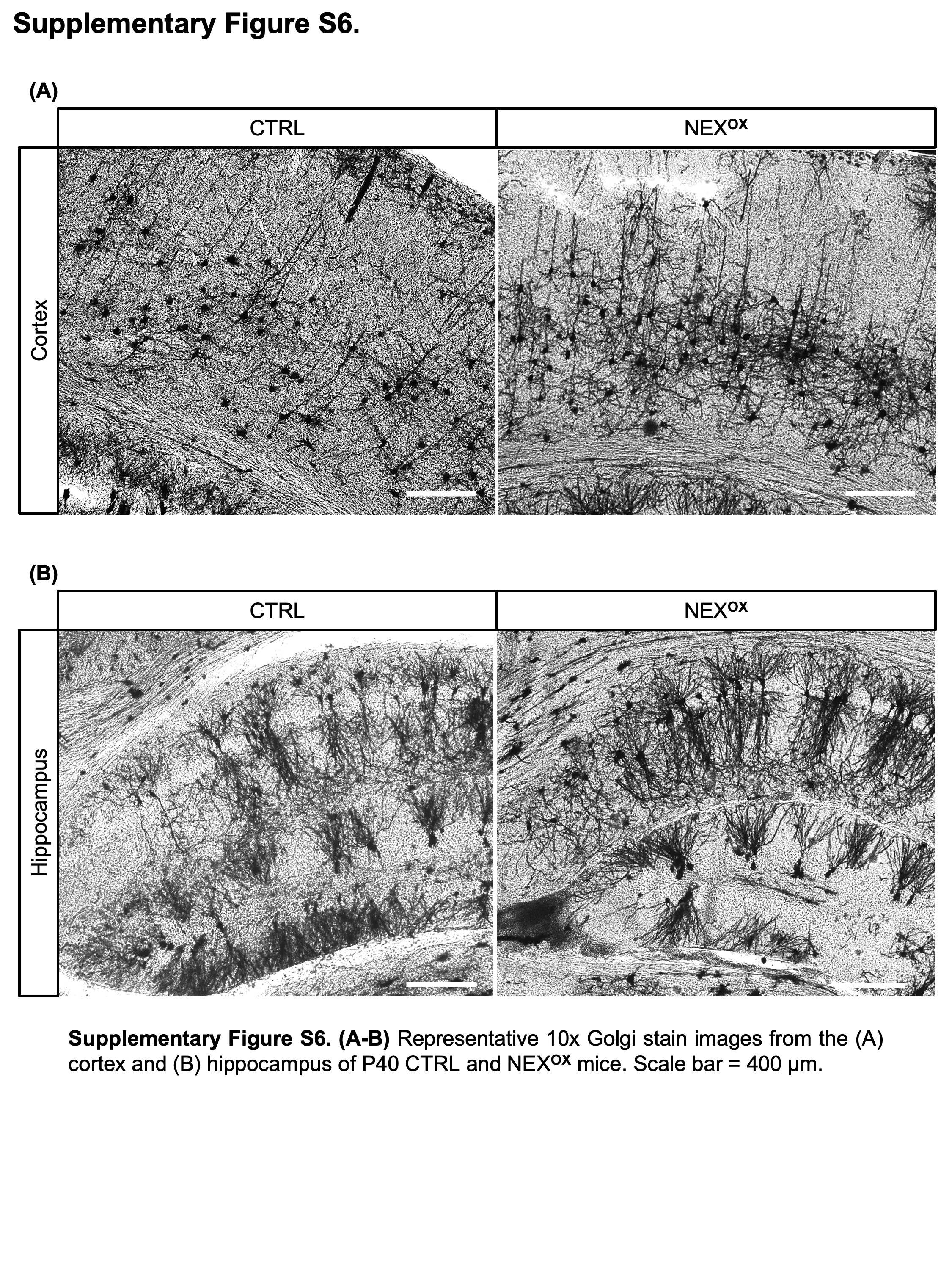

Supplement: Supplementary file 6 [file Image_6.jpeg]
